# Frequency and Geographic Distribution of *gyrA* and *gyrB* Mutations Associated with Fluoroquinolone Resistance in Clinical *Mycobacterium Tuberculosis* Isolates: A Systematic Review

**DOI:** 10.1371/journal.pone.0120470

**Published:** 2015-03-27

**Authors:** Elisea Avalos, Donald Catanzaro, Antonino Catanzaro, Theodore Ganiats, Stephanie Brodine, John Alcaraz, Timothy Rodwell

**Affiliations:** 1 Department of Family and Preventive Medicine, University of California San Diego, La Jolla, California, United States of America; 2 Department of Biological Sciences, University of Arkansas, Fayetteville Arkansas, United States of America; 3 University of California San Diego Medical Center, San Diego, California, United States of America; 4 Graduate School of Public Health, San Diego State University, San Diego, California, United States of America; 5 Division of Global Public Health, University of California San Diego School of Medicine, La Jolla, California, United States of America; London School of Hygiene and Tropical Medicine, UNITED KINGDOM

## Abstract

**Background:**

The detection of mutations in the *gyrA* and *gyrB* genes in the *Mycobacterium tuberculosis* genome that have been demonstrated to confer phenotypic resistance to fluoroquinolones is the most promising technology for rapid diagnosis of fluoroquinolone resistance.

**Methods:**

In order to characterize the diversity and frequency of gyrA and gyrB mutations and to describe the global distribution of these mutations, we conducted a systematic review, from May 1996 to April 2013, of all published studies evaluating Mycobacterium tuberculosis mutations associated with resistance to fluoroquinolones. The overall goal of the study was to determine the potential utility and reliability of these mutations as diagnostic markers to detect phenotypic fluoroquinolone resistance in Mycobacterium tuberculosis and to describe their geographic distribution.

**Results:**

Forty-six studies, covering four continents and 18 countries, provided mutation data for 3,846 unique clinical isolates with phenotypic resistance profiles to fluoroquinolones. The gyrA mutations occurring most frequently in fluoroquinolone-resistant isolates, ranged from 21–32% for D94G and 13–20% for A90V, by drug. Eighty seven percent of all strains that were phenotypically resistant to moxifloxacin and 83% of ofloxacin resistant isolates contained mutations in gyrA. Additionally we found that 83% and 80% of moxifloxacin and ofloxacin resistant strains respectively, were observed to have mutations in the gyrA codons interrogated by the existing MTBDR*sl* line probe assay. In China and Russia, 83% and 84% of fluoroquinolone resistant strains respectively, were observed to have gyrA mutations in the gene regions covered by the MTBDR*sl* assay.

**Conclusions:**

Molecular diagnostics, specifically the Genotype MTBDR*sl* assay, focusing on codons 88–94 should have moderate to high sensitivity in most countries. While we did observe geographic differences in the frequencies of single gyrA mutations across countries, molecular diagnostics based on detection of all gyrA mutations demonstrated to confer resistance should have broad and global utility.

## Introduction

*Mycobacterium tuberculosis* (*Mtb*) is a worldwide public health threat responsible for approximately 8.6 million incident cases of tuberculosis (TB) and an estimated 1.3 million deaths annually [1]. The emergence and increasing prevalence of *Mtb* strains resistant to first and second line antituberculous medications are exacerbating the global TB epidemic [2]. Multidrug resistant (MDR) strains are *Mtb* strains resistant to both rifampicin (RIF) and isoniazid (INH), the most effective first-line drugs. Extensively drug resistant (XDR) *Mtb* strains, are defined as strains with MDR plus resistance to any fluoroquinolone (FQ) and one of the second-line injectable drugs used commonly for treating TB [3]. As of 2012, the World Health Organization (WHO) estimated the global prevalence of MDR-TB to be 3.6% among new TB cases and 20% among recurrent TB cases [1].

As M/XDR-TB rates continue to increase, the development and implementation of rapid diagnostic systems for the detection of microbial resistance to prevent further transmission and promptly implement appropriate drug regimens are needed [4]. Automated liquid culture systems have significantly shortened turnaround times for drug susceptibility tests (DSTs) compared to solid media, but bacteriological assays are technically demanding and still require a sophisticated biosafety environment and approximately 7 to 10 days to complete [4]. Detection of genetic mutations that confer resistance to certain antimicrobial agents represents a more rapid alternative [4]. Currently, the only broadly available commercial assay for the rapid detection of second-line-drug resistance, including FQ resistance, the MTBDR*sl* assay (Hain Lifescience, Nehren, Germany), detects only the most common mutations found in the quinolone resistance determining region (QRDR) of *gyrA [5].*

The main cellular target of FQs in *Mtb* is the DNA gyrase, a type II topoisomerase, which consists of two A and two B subunits encoded by *gyrA* and *gyrB* genes, respectively [2]. The genetic mechanism of resistance to FQs is a result of changes in the DNA gyrase, particularly, mutations in the QRDR of *gyrA* (codons 74 to 113) [6] and *gyrB* (codons 500 to 538) [7]. It has been estimated that roughly 60 to 90% of *Mtb* clinical isolates with FQ resistance have mutations in the QRDR of *gyrA*, particularly in codons 88, 90, 91, and 94 [8–10]. Mutations in *gyrB* have also been associated with FQ resistance, but with lower sensitivity and specificity, and they often co-occur with canonical *gyrA* mutations [11–15] and most often occur in codons 500 and 538 [16]. While most *Mtb* strains with *gyrA* mutations in the QRDR are FQ resistant, nearly all isolates with a wild type QRDR are FQ susceptible. The exceptions are the polymorphisms of *gyrA* at codons 21, 95 and 668 [14, 17, 18], which do not appear to be related to resistance.

FQs have potent *in vitro* activities against *Mtb* [19]. However, FQs are widely used to treat bacterial infections of the respiratory, gastrointestinal, and urinary tract as well as sexually transmitted diseases, further contributing to the increasing levels of FQ resistance in *Mtb* [20, 21]. FQs have proven to be among the most effective second-line anti-mycobacterial drugs [14, 21] and are recommended for the treatment of drug-resistant TB and for persons intolerant of current first-line therapy [17, 22]. While resistance to some of the older generation of FQs has been shown to emerge during treatment of patients infected with FQ-susceptible strains [20], newer generation FQs have become vital in the successful treatment of drug resistant TB [2, 3, 23]. As a result of the promising clinical activity of these newer FQs, the WHO currently recommends levofloxacin or moxifloxacin for the treatment of XDR-TB when ofloxacin resistance is present [24, 25].

In order to characterize the *gyrA* and *gyrB* mutations associated with global phenotypic resistance to the most commonly used FQs in *Mtb* we conducted a systematic review of English language studies from May 1996 to April 2013. The overall goals of the study were to: 1) characterize the diversity and frequency of *gyrA* and *gyrB* mutations in FQ resistant *Mtb* and 2) to describe the global distribution of these mutations to help determine their potential utility and reliability as diagnostic markers for detecting phenotypic FQ resistance in *Mtb*.

## Methods

### Literature Search

A Medline search was conducted of all publications investigating *gyrA* and *gyrB* mutations associated with phenotypic FQ resistance in *Mtb*. The search was restricted to studies published from May 1996 through April 15, 2013, including those studies available online prior to publication. MEDLINE/PubMed key search terms used with the help of Boolean operators (‘and’, ‘or’) were: “tuberculosis,” “fluoroquinolone,” “resistance,” “resistant” “*gyrA*,” “*gyrB*,” “mutation,” “sequence.”

### Study Selection Criteria

Study selection criteria were similar to those described in Georghiou et al. [26]. Studies were included if they met the following predetermined criteria: i) published in English ii) presented original data and iii) assessed drug resistance mutations in clinical *Mtb* strains resistant to FQs (*in vitro* studies were excluded as laboratory generated mutations have been observed to be different from those found in clinical isolates) [27]. Studies were also excluded if they did not mention the specific FQ tested, did not perform or describe details of phenotypic drug susceptibility testing, did not perform sequencing as a method for determining mutations associated with drug resistance. Additionally, studies were excluded if they did not mention the country the clinical isolates originated from or if they listed multiple countries and did not distinguish clinical isolates by country.

### Data Extraction and Entry

The following background variables were collected from the selected publications: author(s), year of publication, geographic origin of clinical strains, the reference strain used, methods for testing phenotypic drug susceptibility and genotypic mutations, MIC levels for each drug, genes sequenced, and loci of genes sequenced. The following mutation information was also recorded: specific gene mutation(s) found, FQ drug utilized for selection, number of resistant and susceptible isolates tested, and number of resistant and susceptible isolates demonstrating a mutation. Data was recorded and compiled using Microsoft Excel (Microsoft, Redmond, WA).

### Data Collation and Cumulative Mutation Frequency Calculations

Data concerning mutations associated with FQ resistance were grouped by gene and stratified by the drug resistance phenotype associated with the mutation. Studies that specifically reported multiple mutations within a gene were also analyzed separately in order to determine the frequency of multiple mutations in genes associated with FQ resistance. Each mutation reported in a resistant *Mtb* isolate was considered independent of all others within and between studies (except where otherwise noted for multiple mutations in the same gene) and recorded as one instance of the mutation in the numerator of the cumulative mutation frequency calculations. Cumulative mutation frequency in *resistant* isolates was calculated as the number of resistant isolates in which the mutation was found, divided by the total number of phenotypically resistant isolates tested across studies. Cumulative mutation frequency in *susceptible* isolates was calculated as the number of susceptible isolates in which the mutation was found, divided by the total number of susceptible isolates tested across studies. As not all studies examined all mutations or all genes associated with resistance, isolates from a study were only included in the denominator of a cumulative frequency mutation calculation for a particular mutation if that mutation could have been detected in that study (i.e. the study sequenced the appropriate section of the gene). In order to accurately assess which gene fragments had been sequenced for each isolate, the exact start and end points of the gene fragments sequenced had to be determined. These endpoints were identified by entering the published primer sequences into the NCBI BLAST (Basic Local Alignment Search Tool) with *Mtb* H37Rv complete genome selected as the reference genome, Accession number NC_000962.3 and mapping the coordinates on *Mtb* H37Rv. Sequence fragments were inferred for articles that did not include primer sequences by using the outermost identified mutations as sequence endpoints. If several primers were included and sequenced fragments overlapped, the final dataset included only the outermost/inclusive primers.

The cumulative mutation frequency tables presented in the main body of the review represent the mutations that reached a frequency threshold, described as the following: 1) Isolates were included if a mutation was observed in at least two studies and reported resistance to at least two FQs with a frequency of at least 1% for any one of the FQs tested; 2) Mutations were excluded from the main tables when the frequencies of the mutation were equal in resistant and susceptible strains. Due to the large number of mutations reported (146 total), this frequency threshold was used to report only the most frequently reported mutations in the main tables. All mutations not meeting the above mentioned criteria, are available in S1 Table.

## Results

### Description of Included Studies

**Fig 1:**
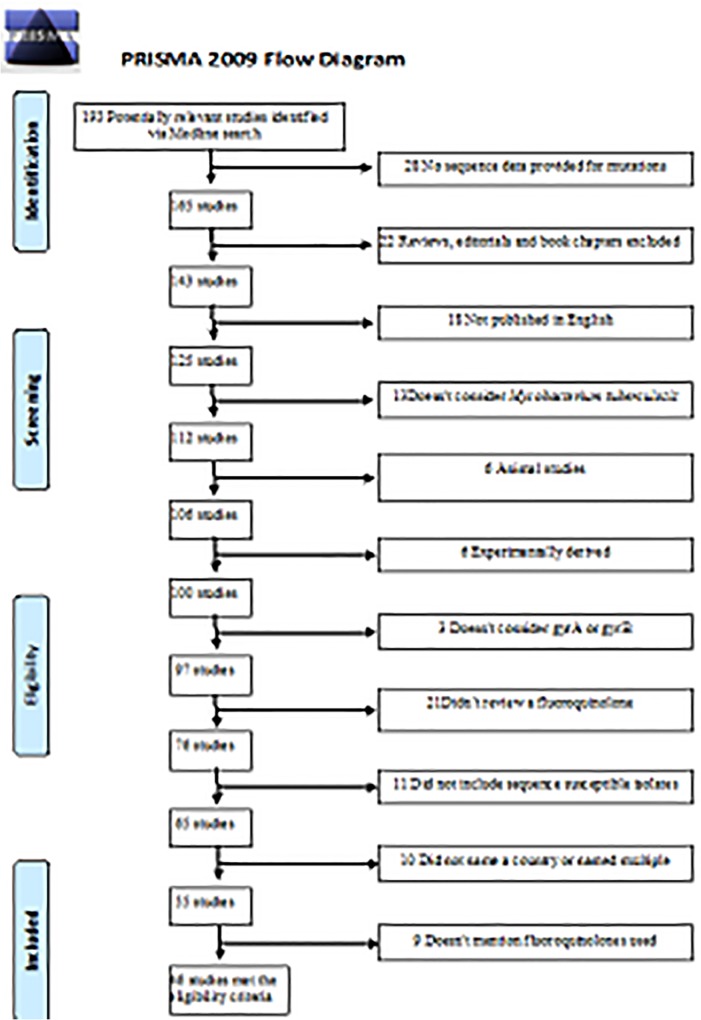
Study Selection Process and Reason for Exclusion of Studies.

Fig. 1 illustrates the study selection and exclusion process utilized for this review. Initial search parameters identified 193 studies published from May, 1996 through April 15, 2013. Forty-six publications met all eligibility criteria and were included in the review [3, 4, 8–10, 12, 15, 18, 20, 22, 27–62]. (PRISMA checklist included in S2 Table).

Of the 46 studies included, the earliest was published in 1996, with 23 (50%) published in the last three years (Table 1). Altogether, mutation data was provided for 3,846 unique clinical *Mtb* isolates with various phenotypic resistance profiles to FQs. The reported geographic origins of these strains were diverse, covering four continents and 18 countries.

**Table 1:** Details of Studies Included in Review and Source of *Mycobacterium tuberculosis* Isolates.

PubMed ID	Author (Year)	# of FLQ Isolates Examined ⁺	Origin of Isolates	Molecular Technique	Clinical Institution(s) Providing Isolates	Year of Collection
23491718	Chernyaeva et al. (2013)	50	Russia	Sequencing	TB Dispensary	2011
23561273	Jnawali et al. (2013)	123	South Korea	PCR & Sequencing	Korea Mycobacterium Resource Center	2009–2010
23019190	Nosova et al. (2013)	68	Russia	Sequencing & TB-BIOCHIP-2	Not Stated	Not Stated
23146281	Poudel et al. (2013)	13	Nepal	PCR & Sequencing	German Nepal Tuberculosis Project	2007–2010
22552454	Chen et al. (2012)	93	China	PCR & Sequencing	Not Stated	2009–2010
22526012	Long et al. (2012)	177	China	PCR & Sequencing	National Tuberculosis Reference Laboratory	Not Stated
22357804	Sirgel et al. (2012)	177	South Africa	Sequencing	Not Stated	2007–2009
22330913	Streicher et al. (2012)	181	South Africa	Sequencing	National Health Laboratory Service	2006–2008
22421328	Suzuki et al. (2012)	59	Japan	PCR & Sequencing	11 Hospitals in Japan	Not Stated
23205246	Tahmasebi et al. (2012)	97	Iran	PCR-SSCP & Sequencing	Mycobacteriology Research Center, Masih Daneshvari Hospital	Not Stated
22553245	Yuan et al. (2012)	58	China	PCR & Sequencing	Jiangxi Chest Hospital	2010–2011
22560167	Zhu et al. (2012)	227	China	PCR & Sequencing	Not Stated	2007–2010
21911575	Ali et al. (2011)	39†	Pakistan	PCR & Sequencing	Aga Khan University Clinical Microbiology Laboratory	2004–2009
22152119	Anand et al. (2011)	39	India	Sequencing	Not Stated	Not Stated
21051549	Ando et al. (2011)	33	Japan	PCR, Sequencing & LiPA	Nine Hospitals in Japan	2002
		17			National Center for Global Health and Medicine	2003–2008
21443804	Cui et al. (2011)	192	China	PCR & Sequencing	Not Stated	2009
21653760	El Sahly et al. (2011)	36	United States	Sequencing	Mycobacteriology Laboratory at Texas Department of State Health Services	2007–2008
21562102	Huang et al. (2011)	74‡	Taiwan	GenoType MTBDRsl & PCR	Various Hospitals	2008–2009
21450523	Hu et al. (2011)	31	China	Sequencing	Local TB Dispensaries	2004–2005
21632897	Kontsevaya et al. (2011)	51	Russia	Sequencing, Pyrosequencing & GenoType MTBDRsl	Various TB Clinics in Samara Region, Russian Federation	2008
21555766	Sekiguchi et al. (2011)	11	Japan	PCR	Not Stated	Not Stated
21623040	Singh et al. (2011)	8	India	PCR & Sequencing	Mycobacterial Repository Centre of the Institute	2004–2008
22115861	Zhao et al. (2011)	125	China	MAS-PCR, PCR-RFLP & Sequencing	Not Stated	Not Stated
20335420	Brossier et al. (2010)	52	France	Sequencing & GenoType MTBDRsl	French Reference Center for Mycobacteria	2005–2009
20573868	Kiet et al. (2010)	62	Vietnam	Sequencing & GenoType MTBDRsl	Pham Ngoc Thach Hospital	2005–2006
20956608	Lau et al. (2010)	71	China	PCR & Sequencing	Queen Mary Hospital and Grantham Hospital	2003–2007
		99				2008–2009
20452372	Yin et al. (2010)	62	China	PCR & Sequencing	Guangdong Chest Hospital	2008–2009
19846642	Bravo et al. (2009)	102	Philippines	PCR & Pyrosequencing	University of the Philippines-Philippine General Hospital	Not Stated
19721073	Duong et al. (2009)	109	Vietnam	Sequencing	Pham Ngoc Thach Hospital	2005–2007
19386845	Hillemann et al. (2009)	106	Germany	Sequencing & GenoType MTBDRsl	National Reference Laboratory	Not Stated
20028780	Perdigao et al. (2009)	26	Portugal	PCR & Sequencing	Hospitals and Laboratories in Lisbon's Health Region	2005
19024017	Antonova et al. (2008)	107	Russia	PCR, Biochip & Sequencing	Not Stated	Not Stated
18559646	Mokrousov et al. (2008)	71	Russia	PCR & Sequencing	St. Petersburg Research Institute of Phthisiopulmonology	2006
18164184	Sun et al. (2008)	110	China	PCR, DHPLC & Sequencing	Beijing Chest Hospital	2002–2004
18544197	van Doorn et al. (2008)	82	Vietnam	PCR, RT-PCR & Sequencing	Pham Ngoc Thach Hospital	2005–2006
17360809	Chan et al. (2007)	250	China	PCR-SSCP/ MPAC & Sequencing	Grantham Hospital and Public Health Laboratory	1994–2004
17934259	Escribino et al. (2007)	18	Spain	PCR & Sequencing	Not Stated	Not Stated
17434825	Sulochana et al. (2007)	118	India	PCR & Sequencing	Not Stated	Not Stated
17412727	Wang et al. (2007)	42	Taiwan	PCR & Sequencing	Tertiary Care Referral Centre	2004–2005
16584301	Kam at al. (2006)	143	China	Sequencing	TB Reference Laboratory, Department of Health	1999–2003
16204341	Huang et al. (2005)	141	Taiwan	PCR & Sequencing	Kaohsiung Veterans General Hospital	1995–2003
15195248	Post et al. (2004)	13	South Africa	Sequencing	Not Stated	Not Stated
12044302	Lee et al. (2002)	100	Singapore	PCR & Sequencing	Central Tuberculosis Laboratory	Not Stated
11796356	Siddiqi et al. (2002)	68	India	PCR & Sequencing	Outpatient hospitals and National Mycobacterial Repository	1995–1998
8737156	Williams et al. (1996)	9	China	PCR & Sequencing	Not Stated	Not Stated
8896523	Xu et al. (1996)	19	United States	PCR & Sequencing	Public Health Research Institute Tuberculosis Center	Not Stated

⁺Does not include reference strain

†Included S95T; not reported here

‡Examined 234 isolates, reported 74

A total of 146 unique mutations were reported relative to the reference H37Rv genome: *gyrA* (76 unique mutations, 34 single mutations and 42 multiple mutations), *gyrB* (28 unique mutations, 25 single mutations and 3 multiple mutations) and *gyrA and gyrB* (42 multiple mutations). We evaluated the DST methods and critical drug concentrations used in each study to define whether a strain was phenotypically resistant or not. Table 2 shows the DST methods and critical concentrations used in each of the included studies and whether or not they conformed to published reference standards. The drug concentrations used in 35 of the 46 (76%) studies conformed to at least one national or international published standard, 4 (9%) studies were conducted in national reference laboratories. The remaining 7 (15%) studies did not document a specific reference laboratory standard.

**Table 2:** Drug Susceptibility Testing (DST) Methods Employed in Publications.

Author	DST Method	Second Generation		Third Generation		Fourth Generation		
		CIPRO	OFL	LEVO	SPAR	GAT	MOX	SITA
Tahmasebi [52] et al.	LJ	2.0⁺	—	—	—	—	—	—
Wang [20] et al.	LJ	2.0⁺	2.0⁺	1.0^‡‡^	—	—	0.5^‡‡^	—
Hu [46] et al.	LJ	2.0⁺	2.0⁺	1.0^‡‡^	—	—	—	—
Chen [53] et al.	LJ	1.0–16.0^††^	2.0⁺–16.0^†^	—	°	0.125–8.0^‡‡^	0.125–16.0^‡‡^	°
Poudel [54] et al.	LJ	—	2.0⁺	—	—	—	—	—
Yuan [55] et al.	LJ	—	2.0⁺	—	—	—	-	—
Williams [31] et al.	LJ	—	2.0⁺	—	—	—	—	—
Jnawali [56]et al.	LJ	—	2.0⁺	—	—	—	—	—
Zhao [41] et al.	LJ	—	2.0⁺	—	—	—	—	—
Brossier [47] et al.	LJ	—	2.0⁺	—	—	—	—	—
Kiet [40] et al.	LJ	—	2.0⁺	—	—	—	—	—
Duong [12] et al.	LJ	—	2.0⁺	—	—	—	—	—
Mokrousov [8] et al.	LJ	—	2.0⁺	—	—	—	—	—
van Doorn [39]et al.	LJ	—	2.0⁺	—	—	—	—	—
Hillemann [9] et al.	LJ/MGIT 960	—	2.0⁺	—	—	—	—	—
Nosova [57] et al.	LJ	—	2.0⁺	2.0⁺⁺	—	0.5⁺⁺	0.5⁺⁺	—
Anand [22] et al.	LJ	—	2.0⁺–4.0^†^	—	—	2.0–5.0^‡‡^	2.0–5.0^‡‡^	—
Chernyaeva [51] et al.	LJ	—	2.0⁺–10.0⁺⁺	—	—	—	—	—
Antonova [29] et al.	LJ	—	2.0⁺, 10.0^†^	—	—	—	—	—
Long [62] et al.	LJ	—	5.0–50.0^†^	2.0–20.0^‡‡^	—	—	—	—
Kam [49] at al.	LJ/MGIT 960	—	0.5^‡^, 1.0^‡^, 2.0⁺, 4.0^†^, 8.0^†^, 16.0^†^	—	—	—	0.5⁺, 1.0^‡‡^, 2.0^‡‡^, 4.0^‡‡^, 8.00^‡‡^, 16.00^‡‡^	—
Sun [27] et al.	LJ	—	0.5^‡^, 1.0^‡^, 2.0⁺, 4.0^†^, 8.0^†^, 10.0^†^, 16.0^†^, 20.0^†^	—	—	—	—	—
Sulochana [38] et al.	LJ	—	8.0^†^	—	—	—	—	—
Chan [36] et al.	LJ	—	—	—	—	—	4.8⁺⁺	—
Siddiqi [33] et al.	LJ	—	—	—	—	—	2.0^‡‡^	—
Perdigao [48]et al. 2007	BACTEC 460	—	2.0⁺	—	—	—	—	—
Zhu [58] et al.	MGIT 960	—	2.0⁺	—	—	—	—	—
Kontsevaya [4] et al.	MGIT 960	—	2.0⁺	—	—	—	2.0^†^	—
Streicher [59] et al.	MGIT 960	—	2.0⁺	—	—	—	—	—
Cui [15] et al.	MGIT 960	—	2.0⁺	—	—	—	—	—
Sirgel [60] et al.	MGIT 960	—	0.5–10.0^††^	-	—	—	0.125–2.0^††^	—
Singh [44] et al.	Middlebrook 7H9	—	8.0^†^, 16.0^†^, 32.0^†^	—	—	—	—	—
Sekiguchi [50] et al.	Middlebrook 7H10	0.5^‡^	—	0.5^‡^	—	0.06^‡^	—	—
Xu [32] et al.	Middlebrook 7H10	2.0⁺	—	—	—	—	—	—
Ali [42] et al.	Middlebrook 7H11	2.0⁺	—	—	—	—	—	—
Huang [45] et al.	Middlebrook 7H11	2.0⁺	2.0⁺	1.0^‡‡^	—	—	—	—
Suzuki [61] et al.	Middlebrook 7H11	6.25–50.0^†^	—	3.13–25.0^‡‡^	1.56–12.5^‡‡^	0.78–6.25^‡‡^	0.78–12.5^‡‡^	0.39–12.5^‡‡^
Escribano [37] et al.	Middlebrook 7H11	16.0^†^	16.0^†^	8.0^‡‡^	—	2.0^‡‡^	4.0^‡‡^	—
Bravo [10] et al.	Middlebrook 7H10	—	2.0⁺	—	—	—	—	—
Lau [18] et al.	Middlebrook 7H10	—	2.0⁺	—	—	—	1.0^‡‡^	—
Post [34] et al.	Middlebrook 7H10	—	2.0⁺	—	—	—	—	—
Huang [35] et al.	Middlebrook 7H11	—	2.0⁺	—	—	—	—	—
Yin [28] et al.	Middlebrook 7H11	—	—	1.0, 10.0^‡‡^	—	—	—	—
El Sahly [43] et al.	Agar proportion indirect susceptibility assay	—	—	—	—	—	0.5^‡‡^	—
Ando [3] et al.	Broth MIC; Egg based Ogawa medium	2.0–16.0^‡‡^	—	2.0–16.0^‡‡^	1.0–8.0^‡‡^	—	—	—
Lee [30] et al.	E-test	—	—	—	—	—	32.0^‡‡^ °	—

CIPRO = Ciprofloxacin, GAT = Gatifloxacin, LEVO = Levofloxacin, MOX = Moxifloxacin, OFL = Ofloxacin, SITA = Sitafloxacin, SPX = Sparfloxacin, NM = MIC not mentioned, LJ = Lowenstein-Jensen

— = Indicates fluoroquinolone not tested in this study

⁺DST conforms to published standard

†DST above published standard

‡DST below published standard

⁺⁺Absolute concentration, not yet validated

††DST range above and below published standard

‡‡No published standard

°In gyrB only

### *gyrA* Mutations Associated with Fluoroquinolone Resistance

**Fig 2:**
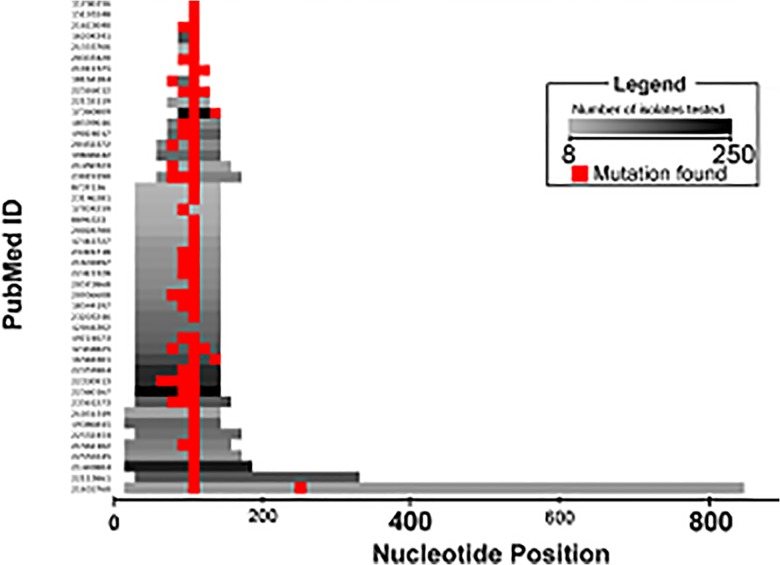
Heat map of Reviewed Studies that Evaluated *gyrA* Gene Mutations in *Mtb*. Heat map of individual papers indicating the number of isolates and the region of the *gyrA* gene studied. The number of isolates testes ranges from 8 (light grey) to 227 (black). Red indicates that a mutation has been found.

Of the 46 papers examined in this review, all 46 studied resistance-associated markers within *gyrA*. Fig. 2 shows the *gyrA* studies as a heat map of the number of isolates evaluated in all 46 studies as well as the locations of the mutations found in *gyrA*. Thirty-four studies sequenced the QRDR of the *gyrA* gene,11 studies sequenced part of the QRDR of the *gyrA* gene; only one study sequenced the entire *gyrA* gene.

Table 3 shows the cumulative frequencies of the most commonly reported mutations in the *gyrA* gene associated with resistance to the primary FQs across publications. Resistance to ofloxacin, levofloxacin and moxifloxacin was studied in the largest number of isolates, but it is important to note that the primary canonical mutations listed in Table 3 appeared to be associated with resistance to all of the clinically relevant FQs, suggesting some level of cross-resistance is likely. Additionally, a subset of studies specifically examined and found evidence of cross resistance associated with these mutations, for example, most moxifloxacin resistant isolates with the A90V mutation (18%) were also resistant to ofloxacin (17%).

**Table 3:** Cumulative Frequencies of the Most Frequently Occurring Mutations within *gyrA* Gene among *Mycobacterium tuberculosis* Isolates Resistant to Fluoroquinolones. Mutations are listed in order of descending frequency.

**Codon**	**Substitution**	**FLQ Tested**	**# Resistant Isolates Examined**	**# Susceptible Isolates Examined**	**# Resistant Isolates with Mutation**	**# Susceptible Isolates with Mutation**	**Frequency of Mutation among Resistant Isolates**	**Frequency of Mutation among Susceptible Isolates**
94	Asp→Gly	OFL	1995	1572	566	0	0.28	0
		MOX	357	540	114	0	0.32	0
		LEVO	412	248	105	0	0.25	0
		CIPRO	334	287	81	0	0.24	0
		GAT	198	91	56	0	0.28	0
		SPX	109	0	23	0	0.21	NA
		SITA	59	0	15	0	0.25	NA
	Asp→Ala	OFL	1995	1572	177	1	0.09	0
		MOX	357	540	43	0	0.12	0
		LEVO	412	248	46	0	0.11	0
		CIPRO	334	287	36	0	0.11	0
		GAT	198	91	26	0	0.13	0
		SPX	109	0	19	0	0.17	NA
		SITA	59	0	10	0	0.17	NA
	Asp→Asn	OFL	1995	1572	122	1	0.06	0
		MOX	357	540	22	1	0.06	0
		LEVO	412	248	22	0	0.05	0
		CIPRO	334	287	28	1	0.08	0
		GAT	198	91	13	1	0.07	0.01
		SPX	109	0	5	0	0.05	NA
		SITA	59	0	5	0	0.08	NA
	Asp→Tyr	OFL	1995	1572	79	0	0.04	0
		MOX	357	540	14	0	0.04	0
		LEVO	412	248	11	0	0.03	0
		CIPRO	334	287	19	0	0.06	0
		GAT	198	91	11	0	0.06	0
		SPX	109	0	6	0	0.06	NA
		SITA	59	0	5	0	0.08	NA
	Asp→His	OFL	1995	1572	21	0	0.01	0
		MOX	357	540	4	0	0.01	0
		LEVO	412	248	3	0	0.01	0
		CIPRO	334	287	1	0	0	0
		GAT	198	91	1	0	0.01	0
	Asp→Val	OFL	1995	1572	4	0	0	0
		MOX	357	540	1	0	0	0
		LEVO	412	248	2	0	0	0
		CIPRO	334	287	2	0	0.01	0
		GAT	198	91	2	0	0.01	0
		SPX	109	0	1	0	0.01	NA
		SITA	59	0	1	0	0.02	NA
90	Ala→Val	OFL	1995	1572	330	4	0.17	0
		MOX	357	540	65	0	0.18	0
		LEVO	412	248	82	0	0.2	0
		CIPRO	334	287	45	0	0.13	0
		GAT	198	91	36	0	0.18	0
		SPX	109	0	16	0	0.15	NA
		SITA	59	0	12	0	0.2	NA
91	Ser→Pro	OFL	1995	1572	84	0	0.04	0
		MOX	357	540	14	0	0.04	0
		LEVO	412	248	9	0	0.02	0
		CIPRO	334	287	18	0	0.05	0
		GAT	198	91	7	0	0.04	0
		SPX	109	0	4	0	0.04	NA
		SITA	59	0	4	0	0.07	NA
88	Gly→Cys	OFL	1982	1504	17	0	0.01	0
		MOX	357	540	5	0	0.01	0
		LEVO	412	248	2	0	0	0
		CIPRO	295	287	1	0	0	0
		GAT	198	91	2	0	0.01	0
		SPX	109	0	1	0	0.01	NA
		SITA	59	0	1	0	0.02	NA
126	Ala→Arg	OFL	1676	1283	4	0	0	0
		MOX	335	523	2	0	0.01	0

CIPRO = Ciprofloxacin, GAT = Gatifloxacin, LEVO = Levofloxacin, MOX = Moxifloxacin, OFL = Ofloxacin, SITA = Sitafloxacin, SPX = Sparfloxacin, NA = Not Applicable

Eighty seven percent of the moxifloxacin resistant isolates and 83% of the ofloxacin resistant isolates had mutations in their *gyrA* genes, with most mutations occurring in codons 88–94 (Table 3, S1 Table and S3 Table). The cumulative frequency of individual mutations associated with FQ resistance was highest for the *gyrA* mutation D94G, ranging from 21–32% in FQ-resistant isolates depending on the specific FQ tested. The *gyrA* A90V mutation was the second most frequent mutation observed in FQ resistant isolates, and was found in 13–20% of FQ-resistant isolates depending on the FQ tested. Across all drugs tested, the *gyrA* mutations G88C and D94V were least frequent (1–2%).

Most importantly, none of the mutations listed in Table 3 occurred in more than a few of the many thousands of FQ susceptible isolates evaluated. Of the 41 studies reporting single A90V mutations, only two studies (n = 4) reported the A90V mutation in FQ susceptible isolates. Two other mutations were reported in susceptible isolates: D94A and D94N, but less than 1% of susceptible isolates contained these mutations, leaving open the possibility these were likely phenotypic DST errors.

### *gyrB* Mutations Associated with Fluoroquinolone Resistance

Eighteen of the 46 (39%) publications included sequence data for *gyrB*. However, overall the *gyrB* mutations have only been evaluated in a few hundred FQ-resistant strains. Mutations of the *gyrB* gene occurred most frequently within ofloxacin resistant isolates (Table 4). The *gyrB* N538D mutation (also reported as N510D in some publications depending on the numbering system used), as well as D500H, T539N and A543V were reported to be rare among ofloxacin-resistant isolates, at frequencies of less than 1%. While the number of susceptible isolates examined for *gyrB* mutations was low, it is important to note than none of them contained mutations listed in Table 4.

**Table 4:** Cumulative Frequencies of the Most Frequently Occurring Mutations within gyrB Gene among *Mycobacterium tuberculosis* Isolates Resistant to Fluoroquinolones. Mutations are listed in order of descending frequency.

Codon	Substitution	FLQ Tested	# Resistant Isolates Examined	# Susceptible Isolates Examined	# Resistant Isolates with Mutation	# Susceptible Isolates with Mutation	Frequency of Mutation among Resistant Isolates	Frequency of Mutation among Susceptible Isolates
538	Asn→Asp	OFL	838	393	3	0	0.00	0.00
		MOX	118	70	2	0	0.02	0.00
		LEVO	315	112	2	0	0.01	0.00
		CIPRO	119	40	1	0	0.01	0.00
		GAT	104	42	1	0	0.01	0.00
500	Asp→His	OFL	838	393	3	0	0.00	0.00
		MOX	118	70	1	0	0.01	0.00
		LEVO	315	112	2	0	0.01	0.00
		GAT	104	42	1	0	0.01	0.00
543	Ala→Val	OFL	536	191	4	0	0.01	0.00
		LEVO	137	40	2	0	0.01	0.00
539	Thr→Asn	OFL	708	239	2	0	0.00	0.00
		LEVO	256	42	2	0	0.01	0.00

CIPRO = Ciprofloxacin, GAT = Gatifloxacin, LEVO = Levofloxacin, MOX = Moxifloxacin, OFL = Ofloxacin, NA = Not Applicable

### Double Mutations in *gyrA* Associated with Fluoroquinolone Resistance

Several studies reported double mutations in *gyrA*, *gyrB* or both *gyrA* and *gyrB*; S3 Table includes double mutations reported within the *gyrA* gene. The most commonly reported double mutations largely included the previously examined A90V mutation. While the cumulative frequencies of *gyrA* double mutations ranged from 1–3% among resistant isolates, no susceptible isolates were reported to contain any of the double mutations, suggesting that although rare, double *gyrA* mutations are highly specific predictors of FQ-resistance.

### Mutations in *gyrA* Associated with Fluoroquinolone Resistance by Country

Table 5 shows the cumulative frequencies of *gyrA* point mutations in FQ resistant isolates by country. The greatest number of studies came from China (n = 13), followed by Russia (n = 5), with all other countries contributing less than four studies each. Both China and Russia reported the *gyrB* mutation D500H in FQ resistant isolates. In China, 85% of mutations reported were found in codons 88–94, whereas 89% of mutations in Russia were in these codons (the remainder of the mutations occurred outside of these codons and in *gyrB*). Of the 18 country-specific studies included in our review, 14 reported mutations in codon 90 (all in A90V) with frequencies ranging from 6% of FQ resistant strains in Iran to 30% of FQ resistant strains in the Philippines. Sixteen countries reported mutations in codon 94. For *gyrA* D94G, the cumulative frequency of the mutation in all FQ resistant strains ranged from 6% in Iran to 56% in South Korea. While A90V and D94G were the most frequently reported mutations overall, four countries reported mutations other than these mutations with higher frequency. In India, the most commonly reported mutation was D94A (20%); in Iran the most commonly reported mutation was D94N (11%); in Portugal the most commonly reported mutation was S91P (42%) and in Spain the most commonly reported mutation was D84G (17%).

**Table 5:** Cumulative Frequencies of Selected Mutations within *gyrA* Gene among *Mycobacterium tuberculosis* Isolates by Country.

Country	Mutation	# Resistant Isolates Examined	# Susceptible Isolates Examined	# Resistant Isolates with Mutation	# Susceptible Isolates with Mutation	Frequency of Mutation among Resistant Isolates	Frequency of Mutation among Susceptible Isolates
China (n = 13)	A90V	1391	1088	253	0	0.18	0.00
	D94G	1391	1088	394	0	0.28	0.00
	D94A	1391	1088	111	1	0.08	0.00
	D94N	1391	1088	117	4	0.08	0.00
	S91P	1391	1088	51	0	0.04	0.00
	D94Y	1391	1088	63	0	0.05	0.00
	D94H	1391	1088	18	0	0.01	0.00
	G88C	1391	1088	3	0	0.00	0.00
	D500H	674	220	3	0	0.00	0.00
France (n = 1)	A90V	24	28	4	0	0.17	0.00
	D94G	24	28	6	0	0.25	0.00
	D94A	24	28	2	0	0.08	0.00
	D94N	24	28	2	0	0.08	0.00
	D94H	24	28	1	0	0.04	0.00
	G88C	24	28	1	0	0.04	0.00
	N538D	24	28	1	0	0.04	0.00
Germany (n = 1)	A90V	32	74	4	0	0.13	0.00
	D94G	32	74	13	0	0.41	0.00
	D94A	32	74	5	0	0.16	0.00
	D94N	32	74	1	0	0.03	0.00
	S91P	32	74	1	0	0.03	0.00
India (n = 4)	A90V	153	158	15	0	0.10	0.00
	D94G	153	158	14	0	0.09	0.00
	D94A	153	158	31	0	0.20	0.00
	D94N	153	158	4	0	0.03	0.00
	S91P	153	158	2	0	0.01	0.00
	D94Y	153	158	2	0	0.01	0.00
Iran (n = 1)	A90V	18	79	1	0	0.06	0.00
	D94G	18	79	1	0	0.06	0.00
	D94N	18	79	2	0	0.11	0.00
Japan (n = 3)	A90V	537	0	93	0	0.17	NA
	D94G	537	0	120	0	0.22	NA
	D94A	537	0	90	0	0.17	NA
	D94N	537	0	33	0	0.06	NA
	S91P	537	0	24	0	0.04	NA
	D94Y	537	0	36	0	0.07	NA
	G88C	537	0	6	0	0.01	NA
Nepal (n = 1)	D94G	13	0	7	0	0.54	NA
	D94A	13	0	2	0	0.15	NA
	D94N	13	0	1	0	0.08	NA
	S91P	13	0	1	0	0.08	NA
	D94Y	13	0	1	0	0.08	NA
	D94H	13	0	1	0	0.08	NA
Pakistan (n = 1)	A90V	39	0	9	0	0.23	NA
	D94G	39	0	14	0	0.36	NA
	D94A	39	0	2	0	0.05	NA
	D94N	39	0	2	0	0.05	NA
	S91P	39	0	1	0	0.03	NA
	D94Y	39	0	5	0	0.13	NA
Philippines (n = 1)	A90V	10	92	3	0	0.30	0.00
	D94G	10	92	3	0	0.30	0.00
Portugal (n = 1)	D94G	52	0	12	0	0.23	NA
	D94A	52	0	16	0	0.31	NA
	S91P	52	0	22	0	0.42	NA
Russia (n = 5)	A90V	364	238	67	3	0.18	0.01
	D94G	364	238	122	0	0.34	0.00
	D94A	364	238	42	0	0.12	0.00
	D94N	364	238	14	0	0.04	0.00
	S91P	364	238	10	0	0.03	0.00
	D94Y	364	238	18	0	0.05	0.00
	D94H	364	238	5	9	0.01	0.04
	G88C	364	238	10	0	0.03	0.00
	D500H	250	143	4	0	0.02	0.00
	N538D	250	143	4	0	0.02	0.00
Singapore (n = 1)	D533A	48	24	1	0	0.02	0.00
South Africa (n = 3)	A90V	280	258	65	0	0.23	0.00
	D94G	280	258	92	0	0.33	0.00
	D94A	280	258	30	0	0.11	0.00
	D94N	280	258	27	0	0.10	0.00
	S91P	280	258	15	0	0.05	0.00
	D94Y	280	258	2	0	0.01	0.00
	G88C	275	250	3	0	0.01	0.00
South Korea (n = 1)	A90V	108	15	16	0	0.15	0.00
	D94G	108	15	60	0	0.56	0.00
	D94A	108	15	2	0	0.02	0.00
	D94N	108	15	3	0	0.03	0.00
	S91P	108	15	9	0	0.08	0.00
	D94Y	108	15	2	0	0.02	0.00
	D94H	108	15	2	0	0.02	0.00
Spain (n = 1)	D84G	35	60	5	0	0.14	0.00
Taiwan (n = 3)	A90V	145	520	15	0	0.10	0.00
	D94G	145	520	51	0	0.35	0.00
	D94A	145	520	3	0	0.02	0.00
	D94N	145	520	5	0	0.03	0.00
	S91P	145	520	2	0	0.01	0.00
	D94Y	145	520	6	0	0.04	0.00
	G88C	145	520	6	0	0.04	0.00
	N538D	56	112	4	9	0.07	0.08
United States (n = 2)	A90V	23	26	4	0	0.17	0.00
	D94G	23	26	3	0	0.13	0.00
	D94A	23	26	1	0	0.04	0.00
	D94N	23	26	3	0	0.13	0.00
	D94Y	23	26	3	0	0.13	0.00
	D94H	23	26	2	0	0.09	0.00
Vietnam (n = 3)	A90V	192	40	37	0	0.19	0.00
	D94G	192	40	48	0	0.25	0.00
	D94A	192	40	20	0	0.10	0.00
	D94N	192	40	3	0	0.02	0.00
	S91P	192	40	2	0	0.01	0.00
	D94Y	192	40	7	0	0.04	0.00
	D94H	192	40	1	0	0.01	0.00

NA = Not applicable, division by zero

## Discussion

From the literature reviewed, it is evident that the QRDR of *gyrA* has been widely studied in FQ resistant *Mtb* isolates; while the remainder of the *gyrA* gene and the *gyrB* gene have been only rarely evaluated. In this review, we found that mutations occurring in the QRDR, specifically in codons 88–94, were found in 85% and 82% of phenotypic moxifloxacin and ofloxacin resistant strains, respectively. These results suggest that *gyrA* mutations in codons 88–94 are likely to be very sensitive markers of phenotypic resistance to FQ drugs in *Mtb* isolates, with high likelihood of cross-resistance to all the major FQs. Only one study included in the review sequenced the entire *gyrA* gene, explaining why very few mutations were reported outside of the QRDR region. The understudied *gyrA* regions may contain mutations that help explain the 15–18% of reported FQ resistant strains that did not appear to have mutations in codons 88–94 of the QRDR of *gyrA*. Additionally the 15–18% of FQ resistant *Mtb* strains with no identified mutation may possess an alternate mechanism of resistance [35, 63, 64]. Low cell wall permeability, efflux-related mechanisms, and drug sequestration or inactivation have been proposed to account for FQ resistance in these isolates [27, 64]. Equally important to the high frequency of the *gyrA* mutations in FQ resistant isolates, is the fact that these mutations occurred in only a few (<1%) FQ susceptible isolates, suggesting that these mutations will have close to 100% specificity as markers of phenotypic FQ resistance. The very few susceptible isolates with QRDR mutations may also have been DST errors as most QRDR mutations (the canonical mutations) have been shown to confer resistance at WHO approved critical concentrations [21].

Mutations in the *Mtb gyrB* gene were also associated with FQ resistance but at a much lower frequency. In this study, these mutations were only evaluated in a few hundred FQ resistant strains and were rare (1–2% of FQ isolates observed). Mutations in *gyrB* typically occur in association with *gyrA* mutations [11–15] and most often occur in codons 500 and 538[16], making it difficult to assess their individual contributions to phenotypic resistance. In a recent study by Malik et al.[21] functional genetic analysis of *gyrB* indicated that certain mutations in *gyrB* confer FQ resistance, however the level and pattern of resistance varied among the different mutations. Nonetheless, the results from their study provide support for the inclusion of mutations in the QRDR of *gyrB* in next generation molecular assays used to detect FQ resistance in *Mtb*. In this review, some *gyrB* mutations did occur independently of *gyrA* mutations which could help explain the phenotypic resistance in isolates that don’t have mutations in the QRDR region of *gyrA*. In our study, the most common *gyrB* mutations occurred in codons 500, 538, 539 and 543. No susceptible isolates were reported to contain *gyrB* mutations, suggesting these rare mutations are highly specific markers of FQ-resistance.

Although rare, *gyrA* double mutations were found to occur in codons 90 and 94. Double mutations suggest *Mtb* may be undergoing adaptive evolution to improve the fitness of the bacteria in response to global FQ treatment [65]. Although the data from this review were limited by the lack of geographical diversity of strains with double mutations, double *gyrA* mutations were never reported in FQ susceptible *Mtb* strains and are likely highly specific markers of FQ resistance in *Mtb*.

In this study, we noted that ofloxacin-resistant clinical isolates were consistently cross-resistant to the newer FQs (eg. moxifloxacin). While there is building evidence to suggest that certain *gyrA* mutations are associated with differential cross resistance to the different FQs, it would appear from our study that many of the canonical *gyrA* mutations should probably be considered broadly cross resistant while evidence of mutation-specific differential resistance is being verified.

The WHO has listed 27 “high burden” TB countries; data from seven of these countries (China, India, Pakistan, Philippines, Russia, South Africa and Vietnam) were included in this review. While several studies have commented on potential geographic differences [2, 15, 28, 40, 42, 45, 46, 51, 66–68] in frequencies of resistance conferring *gyrA* and *gyrB* mutations within and between countries, few attempts have been made to characterize these differences. In our study, we demonstrated that single *gyrA* mutations and resistance to FQs varies geographically. One possible reason for the diversity of mutations between countries may be attributed to different social and geographic transmission environments giving rise to different pressures of natural selection. A second possible reason for this diversity may be attributed to differences in treatment regimens containing FQs, which can result in geographically diverse drug-based selection pressures. Identifying geographical areas with high frequencies of unique mutations may help improve molecular surveillance methods and identify areas of concern for molecular diagnostic assay scale up. However, as long as next generation molecular diagnostics or whole gene/genome approaches are able to detect all of the canonical *gyrA* mutations known to confer resistance, and geographically diverse mutations show the same specificity, the observed spatial diversity of mutations will not decrease sensitivity or specificity of next generation assays.

The WHO Stop TB Program has emphasized the need to strengthen diagnostic testing and the need to develop rapid diagnostics [69]. The only commercial assay for rapid detection of FQ resistance in clinical samples currently is the MTBDR*sl* line probe assay (Hain Lifescience, Nehren, Germany). The MTBDR*sl* assay can detect *Mtb* mutations A90V, S91P, D94A, D94N/Y, D94G, and D94H, with a recently reported pooled sensitivity and specificity of 87% and 97% respectively on direct clinical samples [70]. While we did observe mutations in *gyrA* outside of the codons interrogated by the MTBDR*sl* assay, and in *gyrB* (1–2% of FQ-resistant strains showed single mutations in *gyrB*), our findings indicate that at least 85% and 82% of moxifloxacin and ofloxacin resistant strains, respectively, were observed to contain mutations in the codons interrogated by the MTBDR*sl* assay. This data is consistent with the pooled sensitivity of the MTBDR*sl* assay recently reported in a Cochrane review [71] and suggests that the MTBDR*sl* assay is likely to have good sensitivity for detection of moxifloxacin and ofloxacin resistance globally depending on its ability to detect these mutations in clinical samples. Based on the frequency of QRDR mutations observed in FQ resistant strains in China and Russia (83% and 84% respectively), the MTBDR*sl* assay may have a similar sensitivity in those countries. However, it is important to understand that biases in the collection of strains in the studies from those countries may have contributed to the frequencies observed. This emphasizes the need for representative national and global surveillance of resistance mutations to obtain more reliable estimates of global frequencies of these mutations in order to design next generation molecular diagnostics and optimize global performance.

Recently the WHO Expert Group concluded that based on available evidence, the GenoType MTBDR*sl* assay had a pooled sensitivity and specificity of 84% and 97% respectively. The expert panel determined that while the specificity was sufficient for a “rule-in” test of FQ resistance, it should not be used as a replacement test for conventional phenotypic testing yet [72] due to a high proportion of phenotypic FQ resistant isolates that it appears to be unable to detect. Our review of the global frequencies of *gyrA* mutations in FQ resistant isolates suggests that next generation assays able to detect all of the *gyrA* mutations presented in this review should have sensitivities of at least 87% and 83% for detection of moxifloxacin and ofloxacin resistance respectively, depending on their ability to detect these mutations in clinical samples. Based on our review and previously published work on *gyrA* frequencies by others [16, 73, 74], it seems unlikely that molecular diagnostics based on *gyrA* mutations alone will have global sensitivities exceeding 95%, and may suffer from geographic variability. But it is important to view this limitation in the context of the fact that less than 30–45% of MDR-TB, and likely less FQ resistant TB, is currently being detected by standard phenotypic methods [75]. Existing molecular diagnostics based on detection of QRDR mutations could significantly improve the number of FQ resistant TB cases being detected and treated appropriately.

### Limitations

This study has several limitations. The cumulative frequencies calculated were based on two main assumptions. First, it was assumed that all the mutations reported were independent of each other. If some isolates were misclassified as independent when they were, in fact, not, this could have caused an overestimation in our cumulative frequencies of that specific mutation. Every effort was made to ensure that the isolates and the mutations presented in one study were not also reported in another study. Every manuscript was scrutinized for evidence of the same isolates being reported on and to the best of our knowledge all isolates reported were unique. A second potential source of misclassification error was in our use of the DST results as reported. For example, if an isolate was misclassified as resistant based on faulty DST data, when it was, in fact, susceptible, and it did not have the expected mutation then we would have underestimated the cumulative frequency of that mutation among resistant isolates. To minimize the chances of such misclassification, we excluded manuscripts with no explicit descriptions of their DST methods and clear definitions of what constituted a resistant or susceptible isolate using accepted DST drug concentrations and methodologies. For those studies that did not state which section of a gene was sequenced, this was assumed based on the mutations reported, possibly introducing misclassification bias. Identified “hot spots” were grouped by country (as not all studies reported the city the isolates were collected in) regardless of the year the isolates were collected. Additionally it was assumed that these mutations would remain in the same locations between the time the data were collected and the time of this publication. Moreover, studies reporting from only one country were generalized to the entire country, possibly introducing misclassification bias. Lastly, the exclusion of laboratory generated mutations may have led to the under-reporting of *gyrA* mutations. While laboratory generated mutations and clinical isolates have common features, they also have some key differences. Sun et al. [27]observed mutations occurring in clinical isolates most often did not occur in the laboratory generated mutations. Furthermore, clinical isolates and laboratory generated mutations differed in frequency for various mutation patterns. Thus, while laboratory generated mutations are critical to the understanding of the mechanism of mutations, these mutations do not always accurately reflect the mutations and frequencies of mutations observed in clinical isolates and were therefore excluded from this review of mutations for the purposes of understanding molecular diagnostics for clinical isolates.

## Conclusion

To maximize the sensitivity and specificity of molecular diagnostics based on detection of mutations conferring FQ resistance in *Mtb*, we need an understanding of the frequency and geographic distribution of these mutations. In this review, *gyrA* mutations reported in codons 88–94 appeared to account for at least 82% of phenotypic ofloxacin resistance and 85% of moxifloxacin resistance globally, while g*yrB* mutations and *gyrA* double mutations occurred only rarely. While we did observe geographic differences in the frequencies of specific *gyrA* mutations between countries, it is likely that next generation molecular assays that can detect all of the *gyrA* and *gyrB* mutations documented to confer resistance, will have good sensitivity and specificity globally. Using existing molecular diagnostics to rapidly detect FQ resistance in clinical *Mtb* strains could substantially enhance drug resistance control efforts, with the goal of interruption of disease transmission and ultimately incidence reduction, especially in countries with cross-resistance. While it appears the line probe assay, Genotype MTBDR*sl* should have good sensitivity and specificity for detecting phenotypic FQ resistance globally, future national and international surveillance studies focusing on prevalence of mutations across all of *gyrA* and *gyrB*, could improve design and optimization of next generation molecular diagnostics for detecting FQ resistance.

## Supporting Information

S1 Table List of all mutations not meeting criterion for inclusion. (DOC)

S2 Table PRISMA Checklist. (DOC)

S3 Table Cumulative Frequencies of the Most Frequently Occurring Double Mutations within *gyrA* Gene among *Mycobacterium tuberculosis* Isolates Resistant to Fluoroquinolones. (DOCX)

## References

[1] WHO. Global Tuberculosis Report 2013. Geneva, Switzerland: World Health Organization, 2013.

[2] Al-Mutairi NM, Ahmad S, Mokaddas E. First report of molecular detection of fluoroquinolone resistance-associated gyrA mutations in multidrug-resistant clinical Mycobacterium tuberculosis isolates in Kuwait. BMC research notes. 2011;4:123 10.1186/1756-0500-4-123 21492420 PMC3095995

[3] Ando H, Mitarai S, Kondo Y, Suetake T, Kato S, Mori T, et al Evaluation of a line probe assay for the rapid detection of gyrA mutations associated with fluoroquinolone resistance in multidrug-resistant Mycobacterium tuberculosis. Journal of Medical Microbiology. 2011;60(2):184–8. 10.1099/jmm.0.024729-0 . 21051549

[4] Kontsevaya I, Mironova S, Nikolayevskyy V, Balabanova Y, Mitchell S, Drobniewski F. Evaluation of Two Molecular Assays for Rapid Detection of Mycobacterium tuberculosis Resistance to Fluoroquinolones in High-Tuberculosis and -Multidrug-Resistance Settings. Journal of Clinical Microbiology. 2011;49(8):2832–7. 10.1128/jcm.01889-10 . 21632897 PMC3147752

[5] Pantel A, Petrella S, Matrat S, Brossier F, Bastian S, Reitter D, et al DNA Gyrase Inhibition Assays Are Necessary To Demonstrate Fluoroquinolone Resistance Secondary to gyrB Mutations in Mycobacterium tuberculosis. Antimicrobial Agents and Chemotherapy. 2011;55(10):4524–9. 10.1128/aac.00707-11 . 21768507 PMC3186962

[6] Soudani A, Hadjfredj S, Zribi M, Messaoud T, Masmoudi A, Majed B, et al First report of molecular characterization of fluoroquinolone-resistant Mycobacterium tuberculosis isolates from a Tunisian hospital. Clinical microbiology and infection: the official publication of the European Society of Clinical Microbiology and Infectious Diseases. 2010;16(9):1454–7. 10.1111/j.1469-0691.2009.03087.x 19845696

[7] Pantel A, Petrella S, Veziris N, Brossier F, Bastian S, Jarlier V, et al Extending the Definition of the GyrB Quinolone Resistance-Determining Region in Mycobacterium tuberculosis DNA Gyrase for Assessing Fluoroquinolone Resistance in M. tuberculosis. Antimicrobial Agents and Chemotherapy. 2012;56(4):1990–6. 10.1128/aac.06272-11 . 22290942 PMC3318379

[8] Mokrousov I, Otten T, Manicheva O, Potapova Y, Vishnevsky B, Narvskaya O, et al Molecular characterization of ofloxacin-resistant Mycobacterium tuberculosis strains from Russia. Antimicrobial agents and chemotherapy. 2008;52(8):2937–9. 10.1128/aac.00036-08 . 18559646 PMC2493099

[9] Hillemann D, Ruesch-Gerdes S, Richter E. Feasibility of the GenoType MTBDRsl Assay for Fluoroquinolone, Amikacin-Capreomycin, and Ethambutol Resistance Testing of Mycobacterium tuberculosis Strains and Clinical Specimens. Journal of Clinical Microbiology. 2009;47(6):1767–72. 10.1128/jcm.00081-09 . 19386845 PMC2691112

[10] Bravo LTC, Tuohy MJ, Ang C, Destura RV, Mendoza M, Procop GW, et al Pyrosequencing for Rapid Detection of Mycobacterium tuberculosis Resistance to Rifampin, Isoniazid, and Fluoroquinolones. Journal of Clinical Microbiology. 2009;47(12):3985–90. 10.1128/jcm.01229-09 . 19846642 PMC2786679

[11] An DD, Duyen NTH, Lan NTN, Hoa DV, Ha DTM, Kiet VS, et al Beijing Genotype of Mycobacterium tuberculosis Is Significantly Associated with High-Level Fluoroquinolone Resistance in Vietnam. Antimicrobial Agents and Chemotherapy. 2009;53(11):4835–9. 10.1128/aac.00541-09 . 19721073 PMC2772334

[12] Duong DA, Nguyen THD, Nguyen TNL, Dai VH, Dang TMH, Vo SK, et al Beijing genotype of Mycobacterium tuberculosis is significantly associated with high-level fluoroquinolone resistance in Vietnam. Antimicrobial agents and chemotherapy. 2009;53(11):4835–9. 10.1128/AAC.00541-09 19721073 PMC2772334

[13] Takiff HE, Salazar L, Guerrero C, Philipp W, Huang WM, Kreiswirth B, et al Cloning and nucleotide sequence of Mycobacterium tuberculosis gyrA and gyrB genes and detection of quinolone resistance mutations. Antimicrobial agents and chemotherapy. 1994;38(4):773–80. . 8031045 10.1128/aac.38.4.773 PMC284541

[14] Chakravorty S, Aladegbami B, Thoms K, Lee JS, Lee EG, Rajan V, et al Rapid Detection of Fluoroquinolone-Resistant and Heteroresistant Mycobacterium tuberculosis by Use of Sloppy Molecular Beacons and Dual Melting-Temperature Codes in a Real-Time PCR Assay. Journal of Clinical Microbiology. 2011;49(3):932–40. 10.1128/jcm.02271-10 . 21191047 PMC3067712

[15] Cui Z, Wang J, Lu J, Huang X, Hu Z. Association of mutation patterns in gyrA/B genes and ofloxacin resistance levels in Mycobacterium tuberculosis isolates from East China in 2009. Bmc Infectious Diseases. 2011;11. doi: 78. 10.1186/1471-2334-11-78 . PMC3073916 21443804

[16] Maruri F, Sterling TR, Kaiga AW, Blackman A, van der Heijden YF, Mayer C, et al A systematic review of gyrase mutations associated with fluoroquinolone-resistant Mycobacterium tuberculosis and a proposed gyrase numbering system. Journal of Antimicrobial Chemotherapy. 2012;67(4):819–31. 10.1093/jac/dkr566 . 22279180 PMC3299416

[17] Devasia R, Blackman A, Eden S, Li H, Maruri F, Shintani A, et al High Proportion of Fluoroquinolone-Resistant Mycobacterium tuberculosis Isolates with Novel Gyrase Polymorphisms and a gyrA Region Associated with Fluoroquinolone Susceptibility. Journal of Clinical Microbiology. 2012;50(4):1390–6. 10.1128/jcm.05286-11 . 22189117 PMC3318526

[18] Lau RWT, Ho PL, Kao RYT, Yew WW, Lau TCK, Cheng VCC, et al Molecular Characterization of Fluoroquinolone Resistance in Mycobacterium tuberculosis: Functional Analysis of gyrA Mutation at Position 74. Antimicrobial Agents and Chemotherapy. 2011;55(2):608–14. 10.1128/aac.00920-10 . 20956608 PMC3028802

[19] Alangaden GJ, Manavathu EK, Vakulenko SB, Zvonok NM, Lerner SA. Characterization of fluoroquinolone-resistant mutant strains of Mycobacterium tuberculosis selected in the laboratory and isolated from patients. Antimicrobial agents and chemotherapy. 1995;39(8):1700–3. . 7486904 10.1128/aac.39.8.1700 PMC162811

[20] Wang J-Y, Lee L-N, Lai H-C, Wang S-K, Jan IS, Yu C-J, et al Fluoroquinolone resistance in Mycobacterium tuberculosis isolates: associated genetic mutations and relationship to antimicrobial exposure. The Journal of antimicrobial chemotherapy. 2007;59(5):860–5. 10.1093/jac/dkm061 . 17412727

[21] Malik S, Willby M, Sikes D, Tsodikov OV, Posey JE. New Insights into Fluoroquinolone Resistance in Mycobacterium tuberculosis: Functional Genetic Analysis of gyrA and gyrB Mutations. Plos One. 2012;7(6). 10.1371/journal.pone.0039754 . PMC3386181 22761889

[22] Anand RS, Somasundaram S, Doble M, Paramasivan CN. Docking studies on novel analogues of 8 methoxy fluoroquinolones against GyrA mutants of Mycobacterium tuberculosis. BMC structural biology. 2011;11:47 10.1186/1472-6807-11-47 . 22152119 PMC3298726

[23] Aubry A, Veziris N, Cambau E, Truffot-Pernot C, Jarlier V, Fisher LM. Novel gyrase mutations in quinolone-resistant and-hypersusceptible clinical isolates of Mycobacterium tuberculosis: Functional analysis of mutant enzymes. Antimicrobial Agents and Chemotherapy. 2006;50(1):104–12. 10.1128/aac.50.1.104-112.2006 . 16377674 PMC1346799

[24] Prammananan T, Chaiprasert A, Leechawengwongs M. 8-years experience of fluoroquinolone susceptibility testing of multidrug-resistant Mycobacterium tuberculosis isolates from Siriraj Hospital, Thailand. International journal of antimicrobial agents. 2011;37(1):84–5. 10.1016/j.ijantimicag.2010.09.005 . 21075609

[25] WHO. Anti-Tuberculosis Drug Resistance in the World. Geneva, Switzerland: World Health Organization, 2008.

[26] Georghiou SB, Magana M, Garfein RS, Catanzaro DG, Catanzaro A, Rodwell TC. Evaluation of Genetic Mutations Associated with Mycobacterium tuberculosis Resistance to Amikacin, Kanamycin and Capreomycin: A Systematic Review. Plos One. 2012;7(3). . 10.1371/journal.pone.0033275 PMC3315572 22479378

[27] Sun Z, Zhang J, Zhang X, Wang S, Zhang Y, Li C. Comparison of gyrA gene mutations between laboratory-selected ofloxacin-resistant Mycobacterium tuberculosis strains and clinical isolates. International journal of antimicrobial agents. 2008;31(2):115–21. 10.1016/j.ijantimicag.2007.10.014 . 18164184

[28] Yin X, Yu Z. Mutation characterization of gyrA and gyrB genes in levofloxacin-resistant Mycobacterium tuberculosis clinical isolates from Guangdong Province in China. The Journal of infection. 2010;61(2):150–4. 10.1016/j.jinf.2010.05.001 20452372

[29] Antonova OV, Gryadunov DA, Lapa SA, Kuz'min AV, Larionova EE, Smirnova TG, et al Detection of mutations in Mycobacterium tuberculosis genome determining resistance to fluoroquinolones by hybridization on biological microchips. Bulletin of Experimental Biology and Medicine. 2008;145(1):108–13. 10.1007/s10517-008-0034-5 . 19024017

[30] Lee ASG, Tang LLH, Lim IHK, Wong SY. Characterization of pyrazinamide and ofloxacin resistance among drug resistant Mycobacterium tuberculosis isolates from Singapore. International journal of infectious diseases: IJID: official publication of the International Society for Infectious Diseases. 2002;6(1):48–51. 10.1016/s1201-9712(02)90136-0 . 12044302

[31] Williams KJ, Chan R, Piddock LJ. gyrA of ofloxacin-resistant clinical isolates of Mycobacterium tuberculosis from Hong Kong. The Journal of antimicrobial chemotherapy. 1996;37(5):1032–4. 10.1093/jac/37.5.1032 . 8737156

[32] Xu C, Kreiswirth BN, Sreevatsan S, Musser JM, Drlica K. Fluoroquinolone resistance associated with specific gyrase mutations in clinical isolates of multidrug-resistant Mycobacterium tuberculosis. The Journal of infectious diseases. 1996;174(5):1127–30. . 8896523 10.1093/infdis/174.5.1127

[33] Siddiqi N, Shamim M, Hussain S, Choudhary RK, Ahmed N, Prachee, et al Molecular characterization of multidrug-resistant isolates of Mycobacterium tuberculosis from patients in North India. Antimicrobial agents and chemotherapy. 2002;46(2):443–50. 10.1128/aac.46.2.443-450.2002 . 11796356 PMC127030

[34] Post FA, Willcox PA, Mathema B, Steyn LM, Shean K, Ramaswamy SV, et al Genetic polymorphism in Mycobacterium tuberculosis isolates from patients with chronic multidrug-resistant tuberculosis. The Journal of infectious diseases. 2004;190(1):99–106. 10.1086/421501 . 15195248

[35] Huang TS, Kunin CM, Lee SSJ, Chen YS, Tu HZ, Liu YC. Trends in fluoroquinolone resistance of Mycobacterium tuberculosis complex in a Taiwanese medical centre: 1995–2003. Journal of Antimicrobial Chemotherapy. 2005;56(6):1058–62. 10.1093/jac/dki353 . 16204341

[36] Chan RCY, Hui M, Chan EWC, Au TK, Chin ML, Yip CK, et al Genetic and phenotypic characterization of drug-resistant Mycobacterium tuberculosis isolates in Hong Kong. Journal of Antimicrobial Chemotherapy. 2007;59(5):866–73. 10.1093/jac/dkm054 . 17360809 PMC5404905

[37] Escribano I, Rodriguez C, Llorca B, Garcia-Pachon E, Ruiz M, Royo G. Importance of the efflux pump systems in the resistance of Mycobacterium tuberculosis to Fluoroquinolones and linezolid. Chemotherapy. 2007;53(6):397–401. 10.1159/000109769 . 17934259

[38] Sulochana S, Narayanan S, Paramasivan CN, Suganthi C, Narayanan PR. Analysis of fluoroquinolone resistance in clinical isolates of Mycobacterium tuberculosis from India. Journal of chemotherapy (Florence, Italy). 2007;19(2):166–71. . 10.1179/joc.2007.19.2.166 17434825

[39] van Doorn HR, An DD, de Jong MD, Lan NTN, Hoa DV, Quy HT, et al Fluoroquinolone resistance detection in Mycobacterium tuberculosis with locked nucleic acid probe real-time PCR. The international journal of tuberculosis and lung disease: the official journal of the International Union against Tuberculosis and Lung Disease. 2008;12(7):736–42. . 18544197

[40] Kiet VS, Lan NTN, An DD, Dung NH, Hoa DV, van Vinh Chau N, et al Evaluation of the MTBDRsl test for detection of second-line-drug resistance in Mycobacterium tuberculosis. Journal of clinical microbiology. 2010;48(8):2934–9. 10.1128/JCM.00201-10 20573868 PMC2916598

[41] Zhao L-L, Xia Q, Lin N, Liu Z-G, Zhao X-Q, Wan K-L. Multiplex allele-specific PCR combined with PCR-RFLP analysis for rapid detection of gyrA gene fluoroquinolone resistance mutations in Mycobacterium tuberculosis. Journal of microbiological methods. 2012;88(1):175–8. 10.1016/j.mimet.2011.10.015 22115861

[42] Ali A, Hasan R, Jabeen K, Jabeen N, Qadeer E, Hasan Z. Characterization of Mutations Conferring Extensive Drug Resistance to Mycobacterium tuberculosis Isolates in Pakistan. Antimicrobial Agents and Chemotherapy. 2011;55(12):5654–9. 10.1128/aac.05101-11 . 21911575 PMC3232797

[43] El Sahly HM, Teeter LD, Jost KC Jr, Dunbar D, Lew J, Graviss EA. Incidence of Moxifloxacin Resistance in Clinical Mycobacterium tuberculosis Isolates in Houston, Texas. Journal of Clinical Microbiology. 2011;49(8):2942–5. 10.1128/jcm.00231-11 . 21653760 PMC3147712

[44] Singh M, Jadaun GPS, Ramdas, Srivastava K, Chauhan V, Mishra R, et al Effect of efflux pump inhibitors on drug susceptibility of ofloxacin resistant Mycobacterium tuberculosis isolates. Indian Journal of Medical Research. 2011;133(5):535–40. . 21623040 PMC3121286

[45] Huang W-L, Chi T-L, Wu M-H, Jou R. Performance Assessment of the GenoType MTBDRsl Test and DNA Sequencing for Detection of Second-Line and Ethambutol Drug Resistance among Patients Infected with Multidrug-Resistant Mycobacterium tuberculosis. Journal of Clinical Microbiology. 2011;49(7):2502–8. 10.1128/jcm.00197-11 . 21562102 PMC3147822

[46] Hu Y, Mathema B, Wang W, Kreiswirth B, Jiang W, Xu B. Population-based investigation of fluoroquinolones resistant tuberculosis in rural eastern China. Tuberculosis. 2011;91(3):238–43. 10.1016/j.tube.2011.03.001 . 21450523

[47] Brossier F, Veziris N, Aubry A, Jarlier V, Sougakoff W. Detection by GenoType MTBDRsl Test of Complex Mechanisms of Resistance to Second-Line Drugs and Ethambutol in Multidrug-Resistant Mycobacterium tuberculosis Complex Isolates. Journal of Clinical Microbiology. 2010;48(5):1683–9. 10.1128/jcm.01947-09 . 20335420 PMC2863904

[48] Perdigao J, Macedo R, Malaquias A, Ferreira A, Brum L, Portugal I. Genetic analysis of extensively drug-resistant Mycobacterium tuberculosis strains in Lisbon, Portugal. The Journal of antimicrobial chemotherapy. 2010;65(2):224–7. 10.1093/jac/dkp452 20028780

[49] Kam KM, Yip CW, Cheung TL, Tang HS, Leung OC, Chan MY. Stepwise decrease in moxifloxacin susceptibility amongst clinical isolates of multidrug-resistant Mycobacterium tuberculosis: Correlation with ofloxacin susceptibility. Microbial Drug Resistance-Mechanisms Epidemiology and Disease. 2006;12(1):7–11. 10.1089/mdr.2006.12.7 . 16584301

[50] Sekiguchi J-i, Disratthakit A, Maeda S, Doi N. Characteristic Resistance Mechanism of Mycobacterium tuberculosis to DC-159a, a New Respiratory Quinolone. Antimicrobial Agents and Chemotherapy. 2011;55(8):3958–60. 10.1128/aac.00417-10 . 21555766 PMC3147633

[51] Chernyaeva E, Fedorova E, Zhemkova G, Korneev Y, Kozlov A. Characterization of multiple and extensively drug resistant Mycobacterium tuberculosis isolates with different ofloxacin-resistance levels. Tuberculosis (Edinburgh, Scotland). 2013;93(3):291–5. 10.1016/j.tube.2013.02.005 . 23491718

[52] Tahmasebi P, Farnia P, Sheikholslami F, Velayati A. Rapid identification of extensively and extremely drug resistant tuberculosis from multidrug resistant strains; using PCR-RFLP and PCR-SSCP. Iranian journal of microbiology. 2012;4(4):165–70. . 23205246 PMC3507304

[53] Chen J, Chen Z, Li Y, Xia W, Chen X, Chen T, et al Characterization of gyrA and gyrB mutations and fluoroquinolone resistance in Mycobacterium tuberculosis clinical isolates from Hubei Province, China. The Brazilian journal of infectious diseases: an official publication of the Brazilian Society of Infectious Diseases. 2012;16(2):136–41. . 10.1016/s1413-8670(12)70294-5 22552454

[54] Poudel A, Maharjan B, Nakajima C, Fukushima Y, Pandey BD, Beneke A, et al Characterization of extensively drug-resistant Mycobacterium tuberculosis in Nepal. Tuberculosis (Edinburgh, Scotland). 2013;93(1):84–8. 10.1016/j.tube.2012.10.007 . 23146281

[55] Yuan X, Zhang T, Kawakami K, Zhu J, Li H, Lei J, et al Molecular characterization of multidrug- and extensively drug-resistant Mycobacterium tuberculosis strains in Jiangxi, China. Journal of clinical microbiology. 2012;50(7):2404–13. 10.1128/jcm.06860-11 . 22553245 PMC3405621

[56] Jnawali HN, Hwang SC, Park YK, Kim H, Lee YS, Chung GT, et al Characterization of mutations in multi- and extensive drug resistance among strains of Mycobacterium tuberculosis clinical isolates in Republic of Korea. Diagnostic microbiology and infectious disease. 2013;76(2):187–96. 10.1016/j.diagmicrobio.2013.02.035 . 23561273

[57] Nosova EY, Bukatina AA, Isaeva YD, Makarova MV, Galkina KY, Moroz AM. Analysis of mutations in the gyrA and gyrB genes and their association with the resistance of Mycobacterium tuberculosis to levofloxacin, moxifloxacin and gatifloxacin. Journal of medical microbiology. 2013;62(Pt 1):108–13. 10.1099/jmm.0.046821-0 . 23019190

[58] Zhu C, Zhang Y, Shen Y, Siu GKH, Wu W, Qian X, et al Molecular characterization of fluoroquinolone-resistant Mycobacterium tuberculosis clinical isolates from Shanghai, China. Diagnostic microbiology and infectious disease. 2012;73(3):260–3. 10.1016/j.diagmicrobio.2012.03.025 . 22560167

[59] Streicher EM, Bergval I, Dheda K, Bottger EC, Gey van Pittius NC, Bosman M, et al Mycobacterium tuberculosis population structure determines the outcome of genetics-based second-line drug resistance testing. Antimicrobial agents and chemotherapy. 2012;56(5):2420–7. 10.1128/aac.05905-11 . 22330913 PMC3346650

[60] Sirgel FA, Wiid IJF, van Helden PD. Measuring minimum inhibitory concentrations in mycobacteria. Methods in molecular biology (Clifton, NJ). 2009;465:173–86. 10.1007/978-1-59745-207-6_11 20560078

[61] Suzuki Y, Nakajima C, Tamaru A, Kim H, Matsuba T, Saito H. Sensitivities of ciprofloxacin-resistant Mycobacterium tuberculosis clinical isolates to fluoroquinolones: role of mutant DNA gyrase subunits in drug resistance. International Journal of Antimicrobial Agents. 2012;39(5):435–9. 10.1016/j.ijantimicag.2012.01.007 . 22421328

[62] Long Q, Li W, Du Q, Fu Y, Liang Q, Huang H, et al gyrA/B fluoroquinolone resistance allele profiles amongst Mycobacterium tuberculosis isolates from mainland China. International Journal of Antimicrobial Agents. 2012;39(6):486–9. 10.1016/j.ijantimicag.2012.02.015 . 22526012

[63] Giannoni F, Iona E, Sementilli F, Brunori L, Pardini M, Migliori GB, et al Evaluation of a new line probe assay for rapid identification of gyrA mutations in Mycobacterium tuberculosis. Antimicrobial Agents and Chemotherapy. 2005;49(7):2928–33. 10.1128/aac.49.7.2928-2933.2005 . 15980370 PMC1168684

[64] Louw GE, Warren RM, van Pittius NCG, McEvoy CRE, Van Helden PD, Victor TC. A Balancing Act: Efflux/Influx in Mycobacterial Drug Resistance. Antimicrobial Agents and Chemotherapy. 2009;53(8):3181–9. 10.1128/aac.01577-08 . 19451293 PMC2715638

[65] Sun G, Luo T, Yang C, Dong X, Li J, Zhu Y, et al Dynamic Population Changes in Mycobacterium tuberculosis During Acquisition and Fixation of Drug Resistance in Patients. Journal of Infectious Diseases. 2012;206(11):1724–33. 10.1093/infdis/jis601 . 22984115 PMC3488197

[66] Zhang Z, Lu J, Wang Y, Pang Y, Zhao Y. Prevalence and Molecular Characterization of Fluoroquinolone-Resistant Mycobacterium tuberculosis Isolates in China. Antimicrobial Agents and Chemotherapy. 2014;58(1):364–9. 10.1128/aac.01228-13 . 24165186 PMC3910797

[67] Jeon CY, Calver AD, Victor TC, Warren RM, Shin SS, Murray MB. Use of fluoroquinolone antibiotics leads to tuberculosis treatment delay in a South African gold mining community. International Journal of Tuberculosis and Lung Disease. 2011;15(1):77–83. . 21276301

[68] Surcouf C, Heng S, Pierre-Audigier C, Cadet-Daniel V, Namouchi A, Murray A, et al Molecular detection of fluoroquinolone-resistance in multi-drug resistant tuberculosis in Cambodia suggests low association with XDR phenotypes. Bmc Infectious Diseases. 2011;11 10.1186/1471-2334-11-255 . PMC3224243 21955640

[69] Barnard M, Warren R, Van Pittius NG, van Helden P, Bosman M, Streicher E, et al GenoType MTBDRsl Line Probe Assay Shortens Time to Diagnosis of Extensively Drug-Resistant Tuberculosis in a High-Throughput Diagnostic Laboratory. American Journal of Respiratory and Critical Care Medicine. 2012;186(12):1298–305. 10.1164/rccm.201205-0960OC . 23087027

[70] Feng Y, Liu S, Wang Q, Wang L, Tang S, Wang J, et al Rapid Diagnosis of Drug Resistance to Fluoroquinolones, Amikacin, Capreomycin, Kanamycin and Ethambutol Using Genotype MTBDRsl Assay: A Meta-Analysis. Plos One. 2013;8(2). 10.1371/journal.pone.0055292 . PMC3562191 23383320

[71] Theron G, Peter J, Richardson M, Barnard M, Donegan S, Warren R, et al. The diagnostic accuracy of the GenoType(r) MTBDRsl assay for the detection of resistance to second-line anti-tuberculosis drugs. Cochrane Database of Systematic Reviews; 2014. 10.1002/14651858.CD010705.pub2 PMC4448219 25353401

[72] WHO. The Use of Molecular Line Probe Assay for the Detection of Resistance to Second-Line Anti-Tuberculosis Drugs. Geneva, Switzerland: World Health Organization, 2013.

[73] Rodwell TC, Valafar F, Douglas J, Qian L, Garfein RS, Chawla A, et al Predicting Extensively Drug-Resistant Mycobacterium tuberculosis Phenotypes with Genetic Mutations. Journal of Clinical Microbiology. 2014;52(3):781–9. 10.1128/jcm.02701-13 . 24353002 PMC3957771

[74] Campbell PJ, Morlock GP, Sikes RD, Dalton TL, Metchock B, Starks AM, et al Molecular Detection of Mutations Associated with First- and Second-Line Drug Resistance Compared with Conventional Drug Susceptibility Testing of Mycobacterium tuberculosis. Antimicrobial Agents and Chemotherapy. 2011;55(5):2032–41. 10.1128/aac.01550-10 . 21300839 PMC3088277

[75] WHO. Global Tuberculosis Report 2014. Geneva, Switzerland: World Health Organization, 2014.

