# Supplementary material for: Frequency and Geographic Distribution of gyrA and gyrB Mutations Associated with Fluoroquinolone Resistance in Clinical Mycobacterium Tuberculosis Isolates: A Systematic Review
Source: PLoS One. 2015 Mar 27;10(3):e0120470. doi: 10.1371/journal.pone.0120470 (PMC4376704; doi:10.1371/journal.pone.0120470)
Supplement: S1 Table — (DOC) [file pone.0120470.s001.doc]

**Supplemental Table 1. List of all mutations not meeting criterion for inclusion**

| **Mutation** | **Gene** | **Drug** | **# Resistant Isolates Examined** | **# Susceptible Isolates Examined** | **# Resistant Isolates w/Mutation** | **# Susceptible Isolates w/Mutation** | **Frequency of Mutation Among Resistant Isolates** |
| --- | --- | --- | --- | --- | --- | --- | --- |
|  |  |  |  |  |  |  |  |
|  |  |  |  |  |  |  |  |
| A74S | *gyrA* | MOX | 357 | 540 | 1 | 0 | 0.28% |
|  |  | OFL | 1737 | 1121 | 1 | 0 | 0.06% |
| A74S & D94G | *gyrA* | OFL | 1737 | 1121 | 11 | 0 | 0.63% |
| A74S & D94N | *gyrA* | OFL | 1737 | 1121 | 1 | 0 | 0.06% |
|  |  | CIPRO | 279 | 151 | 1 | 0 | 0.36% |
|  |  | LEVO | 259 | 72 | 1 | 0 | 0.39% |
| A90A | *gyrA* | OFL | 1995 | 1572 | 1 | 0 | 0.05% |
| A90E & T80A | *gyrA* | OFL | 1761 | 1149 | 1 | 0 | 0.06% |
| A90G & D94G & T80A | *gyrA* | OFL | 1761 | 1149 | 1 | 0 | 0.06% |
| A90G & T80A | *gyrA* | OFL | 1761 | 1149 | 0 | 1 | 0.00% |
| A90P & D94G | *gyrA* | OFL | 1995 | 1572 | 1 | 0 | 0.05% |
| A90V & D94C & D94G & D94Y | *gyrA* | OFL | 1995 | 1572 | 1 | 0 | 0.05% |
| A90V & D94G & S91P | *gyrA* | OFL | 1995 | 1572 | 1 | 0 | 0.05% |
| A90V & D94H | *gyrA* | OFL | 1995 | 1572 | 1 | 0 | 0.05% |
| A90V & D94V | *gyrA* | CIPRO | 334 | 287 | 1 | 0 | 0.30% |
|  |  | GAT | 198 | 91 | 1 | 0 | 0.51% |
|  |  | LEVO | 412 | 248 | 1 | 0 | 0.24% |
|  |  | MOX | 357 | 540 | 1 | 0 | 0.28% |
|  |  | SITA | 59 | 0 | 1 | 0 | 1.69% |
|  |  | SPX | 109 | 0 | 1 | 0 | 0.92% |
| A90V & G88A | *gyrA* | OFL | 1982 | 1504 | 2 | 0 | 0.10% |
| A90V & L96P | *gyrA* | CIPRO | 334 | 287 | 1 | 0 | 0.30% |
| A90V & S91A | *gyrA* | OFL | 1995 | 1572 | 1 | 0 | 0.05% |
| D84G | *gyrA* | CIPRO | 279 | 151 | 1 | 0 | 0.36% |
|  |  | GAT | 187 | 91 | 1 | 0 | 0.53% |
|  |  | LEVO | 259 | 72 | 1 | 0 | 0.39% |
|  |  | MOX | 357 | 540 | 1 | 0 | 0.28% |
|  |  | OFL | 1737 | 1121 | 1 | 0 | 0.06% |
| D89G | *gyrA* | MOX | 357 | 540 | 1 | 0 | 0.28% |
|  |  | OFL | 1982 | 1504 | 2 | 0 | 0.10% |
| D89N | *gyrA* | OFL | 1982 | 1504 | 4 | 0 | 0.20% |
| D94A & D94G | *gyrA* | OFL | 1995 | 1572 | 6 | 0 | 0.30% |
| D94A & D94G & S91P | *gyrA* | OFL | 1995 | 1572 | 1 | 0 | 0.05% |
| D94A & S69T | *gyrA* | OFL | 1627 | 1121 | 1 | 0 | 0.06% |
| D94A & S91P | *gyrA* | CIPRO | 334 | 287 | 1 | 0 | 0.30% |
|  |  | GAT | 198 | 91 | 1 | 0 | 0.51% |
|  |  | MOX | 357 | 540 | 1 | 0 | 0.28% |
|  |  | OFL | 1995 | 1572 | 1 | 0 | 0.05% |
| D94A/Y | *gyrA* | OFL | 1995 | 1572 | 1 | 0 | 0.05% |
| D94C | *gyrA* | OFL | 1995 | 1572 | 2 | 0 | 0.10% |
| D94C & D94G & D94N & D94S & D94Y | *gyrA* | OFL | 1995 | 1572 | 1 | 0 | 0.05% |
| D94C & D94G & D94Y | *gyrA* | OFL | 1995 | 1572 | 1 | 0 | 0.05% |
| D94F | *gyrA* | OFL | 1995 | 1572 | 1 | 0 | 0.05% |
| D94G & D111N | *gyrA* | CIPRO | 318 | 151 | 2 | 0 | 0.63% |
| D94G & D94N & D94S | *gyrA* | OFL | 1995 | 1572 | 4 | 0 | 0.20% |
| D94G & D94N & D94Y | *gyrA* | OFL | 1995 | 1572 | 1 | 0 | 0.05% |
| D94G & D94Y | *gyrA* | OFL | 1995 | 1572 | 2 | 0 | 0.10% |
| D94G & S91P | *gyrA* | OFL | 1995 | 1572 | 5 | 0 | 0.25% |
| D94H & S91P | *gyrA* | CIPRO | 334 | 287 | 1 | 0 | 0.30% |
|  |  | GAT | 198 | 91 | 1 | 0 | 0.51% |
|  |  | MOX | 357 | 540 | 1 | 0 | 0.28% |
|  |  | OFL | 1995 | 1572 | 1 | 0 | 0.05% |
| D94N & D94Y | *gyrA* | OFL | 1995 | 1572 | 1 | 0 | 0.05% |
| D94N & G88C | *gyrA* | OFL | 1982 | 1504 | 1 | 0 | 0.05% |
| D94N & G112H | *gyrA* | LEVO | 396 | 112 | 1 | 0 | 0.25% |
|  |  | OFL | 1813 | 1323 | 1 | 0 | 0.06% |
| D94N & S91P | *gyrA* | CIPRO | 334 | 287 | 1 | 0 | 0.30% |
| D94N/G | *gyrA* | OFL | 1995 | 1572 | 2 | 0 | 0.10% |
| D94S | *gyrA* | LEVO | 412 | 248 | 1 | 0 | 0.24% |
|  |  | OFL | 1995 | 1572 | 1 | 0 | 0.05% |
| D94V & G88R | *gyrA* | OFL | 1982 | 1504 | 1 | 0 | 0.05% |
| D94Y & R98L | *gyrA* | OFL | 1843 | 1340 | 1 | 0 | 0.05% |
| D94Y & S91P | *gyrA* | CIPRO | 334 | 287 | 1 | 0 | 0.30% |
| G247S | *gyrA* | MOX | 10 | 26 | 0 | 1 | 0.00% |
| G668D | *gyrA* | OFL | 38 | 20 | 6 | 0 | 15.79% |
| G88A | *gyrA* | LEVO | 412 | 248 | 1 | 0 | 0.24% |
|  |  | OFL | 1982 | 1504 | 4 | 0 | 0.20% |
| G88A & B94Y | *gyrA* | CIPRO | 295 | 287 | 1 | 0 | 0.34% |
|  |  | LEVO | 412 | 248 | 1 | 0 | 0.24% |
|  |  | OFL | 1982 | 1504 | 1 | 0 | 0.05% |
| G88A & H70R | *gyrA* | GAT | 187 | 91 | 1 | 0 | 0.53% |
|  |  | LEVO | 259 | 72 | 1 | 0 | 0.39% |
|  |  | MOX | 357 | 540 | 1 | 0 | 0.28% |
|  |  | OFL | 1737 | 1121 | 1 | 0 | 0.06% |
| H52Q | *gyrA* | OFL | 1474 | 1026 | 1 | 0 | 0.07% |
| H70R | *gyrA* | LEVO | 259 | 72 | 2 | 0 | 0.77% |
|  |  | OFL | 1737 | 1121 | 1 | 0 | 0.06% |
| L109V | *gyrA* | OFL | 1835 | 1340 | 0 | 1 | 0.00% |
| P102H | *gyrA* | MOX | 357 | 540 | 0 | 1 | 0.00% |
|  |  | OFL | 1835 | 1340 | 0 | 1 | 0.00% |
| Q60R | *gyrA* | OFL | 1605 | 1104 | 1 | 0 | 0.06% |
| R68G | *gyrA* | OFL | 1627 | 1121 | 2 | 1 | 0.12% |
| S90P | *gyrA* | OFL | 1995 | 1572 | 1 | 0 | 0.05% |
| S91A | *gyrA* | LEVO | 412 | 248 | 4 | 0 | 0.97% |
| S91L | *gyrA* | OFL | 1995 | 1572 | 1 | 0 | 0.05% |
| S91T | *gyrA* | CIPRO | 334 | 287 | 1 | 0 | 0.30% |
|  |  | GAT | 198 | 91 | 1 | 0 | 0.51% |
|  |  | MOX | 357 | 540 | 1 | 0 | 0.28% |
|  |  | OFL | 1995 | 1572 | 1 | 0 | 0.05% |
| T80S | *gyrA* | MOX | 357 | 540 | 1 | 0 | 0.28% |
|  |  | OFL | 1761 | 1149 | 1 | 0 | 0.06% |
| A471V | *gyrB* | CIPRO | 39 | 0 | 1 | 0 | 2.56% |
| A543T | *gyrB* | OFL | 536 | 191 | 1 | 0 | 0.19% |
| D500A | *gyrB* | OFL | 838 | 393 | 2 | 0 | 0.24% |
| D500H & G509A | *gyrB* | OFL | 838 | 393 | 1 | 0 | 0.12% |
| D500N | *gyrB* | OFL | 838 | 393 | 2 | 0 | 0.24% |
| D533A | *gyrB* | OFL | 838 | 393 | 1 | 0 | 0.12% |
| E419K & T539P | *gyrB* | OFL | 206 | 21 | 1 | 0 | 0.49% |
| E424K | *gyrB* | OFL | 206 | 21 | 4 | 0 | 1.94% |
| E498K | *gyrB* | LEVO | 234 | 70 | 1 | 0 | 0.43% |
|  |  | OFL | 609 | 236 | 1 | 0 | 0.16% |
| E540A | *gyrB* | OFL | 684 | 211 | 1 | 0 | 0.15% |
| E540D | *gyrB* | OFL | 684 | 211 | 1 | 0 | 0.15% |
| E540V | *gyrB* | OFL | 684 | 211 | 1 | 0 | 0.15% |
| G425E | *gyrB* | OFL | 206 | 21 | 1 | 0 | 0.49% |
| G551R | *gyrB* | LEVO | 137 | 40 | 1 | 0 | 0.73% |
|  |  | OFL | 486 | 191 | 1 | 0 | 0.21% |
| G551R & T539N | *gyrB* | LEVO | 137 | 40 | 1 | 0 | 0.73% |
|  |  | OFL | 486 | 191 | 1 | 0 | 0.21% |
| G570R | *gyrB* | MOX | 10 | 26 | 0 | 1 | 0.00% |
| K679R | *gyrB* | MOX | 10 | 26 | 0 | 1 | 0.00% |
| Q577H | *gyrB* | OFL | 254 | 44 | 1 | 0 | 0.39% |
| R485H | *gyrB* | GAT | 38 | 30 | 0 | 1 | 0.00% |
|  |  | LEVO | 38 | 30 | 0 | 1 | 0.00% |
|  |  | MOX | 38 | 30 | 0 | 1 | 0.00% |
|  |  | OFL | 339 | 158 | 0 | 1 | 0.00% |
| R485L | *gyrB* | OFL | 339 | 158 | 1 | 0 | 0.29% |
| S434A | *gyrB* | OFL | 206 | 21 | 1 | 0 | 0.49% |
| S486F | *gyrB* | OFL | 472 | 186 | 1 | 1 | 0.21% |
| S540L | *gyrB* | OFL | 684 | 211 | 2 | 0 | 0.29% |
| T539P | *gyrB* | OFL | 708 | 239 | 1 | 0 | 0.14% |
| A90V & D500A | *gyrA/gyrB* | OFL | 838 | 393 | 1 | 0 | 0.12% |
| A90V & D500N | *gyrA/gyrB* | OFL | 838 | 393 | 1 | 0 | 0.12% |
| A90V & D94A & N538T | *gyrA/gyrB* | OFL | 838 | 393 | 1 | 0 | 0.12% |
| A90V & D94A & D94G & S91P & N538T | *gyrA/gyrB* | OFL | 838 | 393 | 1 | 0 | 0.12% |
| A90V & E498K | *gyrA/gyrB* | LEVO | 234 | 70 | 1 | 0 | 0.43% |
|  |  | OFL | 609 | 236 | 1 | 0 | 0.16% |
| A90V & G551R | *gyrA/gyrB* | LEVO | 137 | 40 | 3 | 0 | 2.19% |
|  |  | OFL | 486 | 191 | 5 | 0 | 1.03% |
| A90V & N538T | *gyrA/gyrB* | OFL | 838 | 393 | 1 | 0 | 0.12% |
| A90V & R485C | *gyrA/gyrB* | OFL | 339 | 158 | 1 | 0 | 0.29% |
| A90V & T539A | *gyrA/gyrB* | LEVO | 256 | 42 | 1 | 0 | 0.39% |
|  |  | OFL | 708 | 239 | 1 | 0 | 0.14% |
| A90V & T539N | *gyrA/gyrB* | CIPRO | 98 | 0 | 2 | 0 | 2.04% |
|  |  | GAT | 59 | 0 | 2 | 0 | 3.39% |
|  |  | LEVO | 256 | 42 | 2 | 0 | 0.78% |
|  |  | MOX | 59 | 0 | 2 | 0 | 3.39% |
|  |  | OFL | 708 | 239 | 2 | 0 | 0.28% |
|  |  | SITA | 59 | 0 | 2 | 0 | 3.39% |
|  |  | SPX | 59 | 0 | 2 | 0 | 3.39% |
| A90V & T539P | *gyrA/gyrB* | OFL | 708 | 239 | 1 | 0 | 0.14% |
| D94A & A543T | *gyrA/gyrB* | LEVO | 137 | 40 | 6 | 0 | 4.38% |
|  |  | OFL | 536 | 191 | 6 | 0 | 1.12% |
| D94A & D500N | *gyrA/gyrB* | OFL | 838 | 393 | 1 | 0 | 0.12% |
| D94A & E424K | *gyrA/gyrB* | OFL | 206 | 21 | 1 | 0 | 0.49% |
| D94A & E481Q & D483H | *gyrA/gyrB* | OFL | 206 | 21 | 1 | 0 | 0.49% |
| D94A & E540D | *gyrA/gyrB* | OFL | 684 | 211 | 1 | 0 | 0.15% |
| D94A & I458M | *gyrA/gyrB* | OFL | 206 | 21 | 1 | 0 | 0.49% |
| D94A & N538K | *gyrA/gyrB* | OFL | 838 | 393 | 1 | 0 | 0.12% |
| D94A & N538T | *gyrA/gyrB* | OFL | 838 | 393 | 2 | 0 | 0.24% |
| D94A & N538I | *gyrA/gyrB* | LEVO | 314 | 112 | 2 | 0 | 0.64% |
|  |  | OFL | 838 | 393 | 2 | 0 | 0.24% |
| D94A & T539P | *gyrA/gyrB* | OFL | 708 | 239 | 2 | 0 | 0.28% |
| D94G & A543V | *gyrA/gyrB* | LEVO | 137 | 40 | 2 | 0 | 1.46% |
|  |  | OFL | 536 | 191 | 6 | 0 | 1.12% |
| D94G & D414K | *gyrA/gyrB* | OFL | 206 | 21 | 1 | 0 | 0.49% |
| D94G & D414P | *gyrA/gyrB* | OFL | 206 | 21 | 1 | 0 | 0.49% |
| D94G & E424K | *gyrA/gyrB* | OFL | 206 | 21 | 4 | 0 | 1.94% |
| D94G & E522Q | *gyrA/gyrB* | OFL | 838 | 393 | 3 | 0 | 0.36% |
| D94G & G551R | *gyrA/gyrB* | OFL | 486 | 191 | 1 | 0 | 0.21% |
| D94G & N538T | *gyrA/gyrB* | OFL | 838 | 393 | 1 | 0 | 0.12% |
| D94G & R485G | *gyrA/gyrB* | OFL | 339 | 158 | 1 | 0 | 0.29% |
| D94G & S486F | *gyrA/gyrB* | CIPRO | 98 | 0 | 1 | 0 | 1.02% |
|  |  | GAT | 97 | 30 | 1 | 0 | 1.03% |
|  |  | LEVO | 97 | 30 | 1 | 0 | 1.03% |
|  |  | MOX | 97 | 30 | 1 | 0 | 1.03% |
|  |  | SITA | 59 | 0 | 1 | 0 | 1.69% |
|  |  | SPX | 59 | 0 | 1 | 0 | 1.69% |
| D94N & A543V | *gyrA/gyrB* | LEVO | 137 | 40 | 2 | 0 | 1.46% |
|  |  | OFL | 536 | 191 | 2 | 0 | 0.37% |
| D94N & D500N | *gyrA/gyrB* | OFL | 838 | 393 | 1 | 0 | 0.12% |
| D94N & E419K & E424K & R460K | *gyrA/gyrB* | OFL | 206 | 21 | 1 | 0 | 0.49% |
| D94N & G551R | *gyrA/gyrB* | LEVO | 137 | 40 | 1 | 0 | 0.73% |
|  |  | OFL | 486 | 191 | 1 | 0 | 0.21% |
| D94N & N538K | *gyrA/gyrB* | OFL | 838 | 393 | 1 | 0 | 0.12% |
| D94N & N538S | *gyrA/gyrB* | OFL | 838 | 393 | 1 | 0 | 0.12% |
| D94N & V461A | *gyrA/gyrB* | OFL | 206 | 21 | 1 | 0 | 0.49% |
| D94V & N538T | *gyrA/gyrB* | OFL | 838 | 393 | 1 | 0 | 0.12% |
| D94Y & E419K | *gyrA/gyrB* | OFL | 206 | 21 | 1 | 0 | 0.49% |
| G88A & G509C | *gyrA/gyrB* | LEVO | 315 | 112 | 2 | 0 | 0.63% |
|  |  | OFL | 830 | 333 | 2 | 0 | 0.24% |
| S91P & N464S | *gyrA/gyrB* | OFL | 206 | 21 | 1 | 0 | 0.49% |
| S91P & D500N | *gyrA/gyrB* | OFL | 838 | 393 | 1 | 0 | 0.12% |

CIPRO = Ciprofloxacin, GAT = Gatifloxacin, LEVO = Levofloxacin, MOX = Moxifloxacin, OFL = Ofloxacin, SITA=Sitafloxacin, SPX=Sparfloxacin
