# Supplementary material for: Frequency and Geographic Distribution of gyrA and gyrB Mutations Associated with Fluoroquinolone Resistance in Clinical Mycobacterium Tuberculosis Isolates: A Systematic Review
Source: PLoS One. 2015 Mar 27;10(3):e0120470. doi: 10.1371/journal.pone.0120470 (PMC4376704; doi:10.1371/journal.pone.0120470)
Supplement: S3 Table — (DOCX) [file pone.0120470.s003.docx]

**Supplemental Table 3. Cumulative Frequencies of the Most Frequently Occurring Double Mutations within *gyrA* Gene among *Mycobacterium tuberculosis* Isolates Resistant to Fluoroquinolones**

| **Mutation 1** | **Mutation 2** | **FLQ Tested** | **# Resistant Isolates Examined** | **# Susceptible Isolates Examined** | **# Resistant Isolates with Mutation** | **# Susceptible Isolates with Mutation** | **Frequency of Mutation among Resistant Isolates** | **Frequency of Mutation among Susceptible Isolates** |
| --- | --- | --- | --- | --- | --- | --- | --- | --- |
|  |  |  |  |  |  |  |  |  |
|  |  |  |  |  |  |  |  |  |
| A90V | D94G | OFL | 1995 | 1572 | 53 | 0 | 0.03 | 0.00 |
|  |  | MOX | 357 | 540 | 4 | 0 | 0.01 | 0.00 |
|  |  | LEVO | 412 | 248 | 1 | 0 | 0.00 | 0.00 |
|  |  | GAT | 198 | 91 | 1 | 0 | 0.01 | 0.00 |
| A90V | S91P | OFL | 1995 | 1572 | 17 | 0 | 0.01 | 0.00 |
|  |  | MOX | 357 | 540 | 1 | 0 | 0.00 | 0.00 |
|  |  | LEVO | 412 | 248 | 2 | 0 | 0.00 | 0.00 |
|  |  | CIPRO | 334 | 287 | 3 | 0 | 0.01 | 0.00 |
|  |  | GAT | 198 | 91 | 2 | 0 | 0.01 | 0.00 |
|  |  | SPX | 109 | 0 | 1 | 0 | 0.01 | NA |
|  |  | SITA | 59 | 0 | 1 | 0 | 0.02 | NA |
| A90V | D94A | OFL | 1995 | 1572 | 8 | 0 | 0.00 | 0.00 |
|  |  | MOX | 357 | 540 | 1 | 0 | 0.00 | 0.00 |
|  |  | LEVO | 412 | 248 | 5 | 0 | 0.01 | 0.00 |
|  |  | CIPRO | 334 | 287 | 3 | 0 | 0.01 | 0.00 |
|  |  | GAT | 198 | 91 | 2 | 0 | 0.01 | 0.00 |
|  |  | SPX | 109 | 0 | 1 | 0 | 0.01 | NA |
|  |  | SITA | 59 | 0 | 1 | 0 | 0.02 | NA |
| A90V | D94N | OFL | 1995 | 1572 | 10 | 0 | 0.01 | 0.00 |
|  |  | LEVO | 412 | 540 | 1 | 0 | 0.00 | 0.00 |
|  |  | CIPRO | 334 | 287 | 1 | 0 | 0.00 | 0.00 |
| D94G | D94N | OFL | 1995 | 1572 | 5 | 0 | 0.00 | 0.00 |
|  |  | MOX | 357 | 540 | 3 | 0 | 0.01 | 0.00 |
|  |  | LEVO | 412 | 248 | 1 | 0 | 0.00 | 0.00 |
|  |  | GAT | 198 | 91 | 1 | 0 | 0.01 | 0.00 |
| A90V | P102H | OFL | 1835 | 1340 | 3 | 0 | 0.00 | 0.00 |
|  |  | MOX | 357 | 540 | 2 | 0 | 0.01 | 0.00 |
| D94A | D94N | OFL | 1995 | 1572 | 3 | 0 | 0.00 | 0.00 |
|  |  | MOX | 357 | 540 | 2 | 0 | 0.01 | 0.00 |
| A90V | D94Y | OFL | 1995 | 1572 | 2 | 0 | 0.00 | 0.00 |
|  |  | MOX | 357 | 540 | 1 | 0 | 0.00 | 0.00 |
|  |  | LEVO | 412 | 248 | 1 | 0 | 0.00 | 0.00 |
|  |  | GAT | 198 | 91 | 1 | 0 | 0.01 | 0.00 |

CIPRO = Ciprofloxacin, GAT = Gatifloxacin, LEVO = Levofloxacin, MOX = Moxifloxacin, OFL = Ofloxacin, SITA=Sitafloxacin, SPX=Sparfloxacin, NA=Not applicable
